# Basal forebrain cholinergic input mediates adaptive attention allocation to enhance olfactory discrimination

**DOI:** 10.1371/journal.pbio.3003374

**Published:** 2025-09-16

**Authors:** Rahul Garg, Qiang Qiu, C. Ron Yu

**Affiliations:** 1 Graduate School of Stowers Institute for Medical Research, Kansas City, Missouri, United States of America; 2 Stowers Institute for Medical Research, Kansas City, Missouri, United States of America; 3 Department of Neurosciences, Case Western Reserve University School of Medicine, Cleveland, Ohio, United States of America; 4 Department of Physiology and Cell Biology, University of Kansas Medical Center, Kansas City, Missouri, United States of America; Okinawa Institute of Science and Technology Graduate University: Okinawa Kagaku Gijustu Daigakuin Daigaku, JAPAN

## Abstract

By selectively amplifying relevant sensory input, animals efficiently allocate limited cognitive resources to improve decision-making. Allocation of attention is aligned with behavioral goals and adaptive to cognitive demand, but the circuit mechanisms remain unclear. Here, we identify an attention circuit for odor processing where cholinergic neurons in the horizontal nucleus of the diagonal band provide top–down control through inhibitory dopaminergic short-axon cells in the mouse olfactory bulb. Attentional cue triggered cholinergic activity provides preparatory disinhibition of olfactory sensory axons to enhance response to reward-associated odors and improves decision-making. Preparatory, but not reward-dependent, cholinergic activity is disengaged in proficient animals when the task becomes routine, underlying a trade-off between proficiency and attention engagement. Direct manipulation of the disinhibitory circuit reinstates attentional effect without eliciting general arousal. A computational model of the circuit recapitulates the dynamic change in attention responses and illustrates a two-stage adaptation that efficiently allocates cognitive resources.

## Abstract 2

Animals can use cues to initiate task preparation and amplify relevant sensory input, but the underlying circuits are poorly understood. This study describes a circuit whereby an attentional cue triggers cholinergic forebrain activity, enhancing olfactory responses, which is deactivated as animals become task-proficient.

## Introduction

Efficient use of limited cognitive resources is vital for survival. Attention, through both external (bottom–up) and goal-directed (top–down) processes, controls perception and behavioral responses [1–6]. Driven by external stimuli, bottom–up processes heighten the arousal state and direct attention towards the source of stimulation [7–9]. Top–down attention, on the other hand, prioritizes information relevant to a task and filters out less relevant sensory inputs [10–12].

Attention engagement is not uniform; it varies according to the cognitive demands of the task at hand. A proposed “law of effort” suggests that the brain opts for the least demanding strategy available when multiple options exist [13]. As one becomes skilled in a task and the response becomes routine, the demand for attention diminishes. These adaptive changes in attention allocation are prominent in discrimination learning and reward-driven behaviors, playing a crucial role in optimizing sensory salience and decision-making. However, the neural mechanisms that drive task-dependent modulation of attention and their integration with reward learning remain unclear.

To address these questions, it is imperative to identify the neural circuits underlying attention allocation and explore how experience shapes the selective modulation of sensory inputs during discrimination learning and across task demands. Tonic acetylcholine release from the cholinergic basal forebrain has been shown to mediate arousal and influence attention [14,15]. Rapid changes in cholinergic modulation have been observed during cortical processing of conditioned stimuli and modulation of sensory perception [16–18]. Delineating the circuit that enables selective modulation, however, is challenging because the specific effect of attention is often entangled with arousal, working memory, and reward systems [19–21]. This complexity masks the unique contribution of attention and hinders a clear understanding of neural circuitry involved. Moreover, while pharmacological intervention and lesion studies of cholinergic system have been explored [22–24], these methods lack specificity and impact multiple neural processes simultaneously. Given these complexities, simple attention tasks that build on temporal expectation, such as those employing fixed foreperiod, provide significant advantages [25,26]. Fixed foreperiod creates a stable and predictable temporal framework that not only enhances experimental control but also mimics real-life scenarios where anticipation of a known event requires sustained attention. By minimizing the cognitive load and extraneous variables associated with more complex paradigms, these tasks allow for a clearer isolation of the neural mechanisms underlying attention.

Here, we establish a cued-attention odor discrimination task to determine modulation of sensory response and behavioral performance. The olfactory bulb (OB) glomerular layer is the first stage of odor information processing. The accessibility of this circuit permits an assessment of how early sensory processing stages are influenced by both bottom–up and top–down mechanisms. OB responses are modulated by periglomerular interneurons and centrifugal feedback [27–29], but it is not known whether the circuit is engaged in a task-dependent manner. We identify an OB circuit motif that receives basal forebrain cholinergic innervation and controls the strength of olfactory input. We demonstrate that differential activation of cholinergic neurons supports valence-specific modulation of glomerular response. The level of engagement of these cholinergic neurons is adaptive and underlies attention associated improvement in decision-making. Through specific manipulation of the circuit, we show that attentional effect can take place without global arousal. Our results demonstrate that the basal forebrain controls resource allocation to optimize attention modulation.

## Results

### Attention reduces behavioral uncertainty in an olfactory task

**Fig 1:**
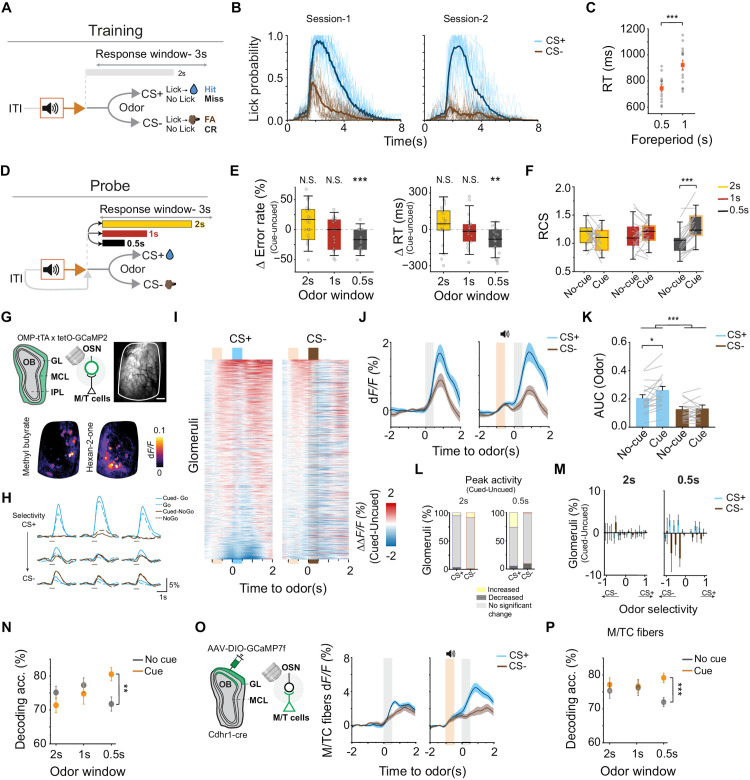
Attention reduces decision uncertainty under high cognitive demand. (A) Schematic of cued- attention odor discrimination training paradigm. Mice were trained to discriminate forthcoming odors following an auditory cue presentation. (B) Licking probability across time. Odor delivered from *t* = 0 to 2 s. (C) Reaction time (RT) for two fixed foreperiods tested during training. ***P < 0.001, one-way repeated measures ANOVA followed by Tukey’s post hoc test (*n* = 14 animals). (D) Schematic of cued- attention odor discrimination paradigm in the test phase. Mice trained to discriminate forthcoming odors following an auditory cue presentation. The cue was omitted in 50% of the trials during test session. Inter-trial interval (ITI) varied between 15 and 45 s. Odor delivery varied between 0.5 and 2 s. (E) Error rate difference (left) and RT change (right) between cued and un-cued odor discrimination trials (*n* = 19 animals). Bars represent mean ± SEM, horizontal lines denote median. The dotted line represents no difference between trials. N.S. (not significant), **P < 0.01, ***P < 0.001, one sample *t* test. (F) Pairwise comparison of rate correct scores (RCS) for cued and un-cued trials. ***P < 0.001, two-way repeated measures ANOVA followed by Tukey’s post hoc test. (G) Top: Schematic and an example imaging plane above dorsal olfactory bulb of *OMP-tTA; tetO-GCaMP2* mouse, scale bar 200 µm. Bottom: Patterns of odor-evoked glomerular activity by two odors. (H) Example activity across trials in individual odor associated glomeruli. (I) Difference of activity between cued and un-cued trials (*ΔΔF*/*F*) for each glomerulus (*n* = 1,066 glomeruli from 19 animals). (J) Average glomerular activity trace for un-cued (left) and cued odor stimulation (right). Orange shading shows cue presentation. Gray shading shows odor delivery. Data are mean ± standard error. (K) Area under the curve (AUC) for odor period (*t* = 0 to 0.5 s). *P < 0.05, ***P < 0.001, two-way repeated measures ANOVA followed by Tukey’s post hoc test. (L) Fraction of glomeruli with significant changes with cued-odor. Yellow: increased, dark grey: decreased, light grey: unchanged. (M) Bar plots show the percentage of glomeruli exhibiting a change in response for cued vs. un-cued trials. Positive values on the *x*-axis indicate greater selectivity for CS+ odors. (N) Accuracy in decoding odor identity based on the glomerular activity for cued (orange) and un-cued (gray) trials. **P < 0.01, two-way repeated measures ANOVA followed by Tukey’s post hoc test. (O) Left: Strategy to image MTC dendrites the glomeruli using GCaMP7F. Right: MTC dendritic activity trace for un-cued (left) and cued odor delivery (right; *n* = 19). (P) Same as (N) but for MTC dendritic activities. ***P < 0.001, two-way repeated measures ANOVA followed by Tukey’s post hoc test. Data for this figure are provided in S1 Data.

In the cued-attention odor discrimination paradigm, we trained mice to discriminate between a pair of odor stimuli that predicted reward and punishment (CS+ and CS−; Fig 1A). An auditory cue, lacking direct relevance to odor identity or value, was presented prior to odor onset as an attention signal. After two training sessions, mice exhibited an increase in decision accuracies for discriminating odors and reduced anticipatory licking during the foreperiod between the odor and auditory cue (Figs 1B and S1A–S1C). We first tested how foreperiod duration affected reaction time (RT). A 0.5 s delay between cue and odor shortened RTs compared to a 1-s delay, indicating stronger state of readiness with shorter foreperiod (Fig 1C; *q*(13) = 8.58, *p* < 0.0001).

We next assessed how the attention cue modulates performance in trials of varying difficulty. In probe sessions, we randomly omitted the auditory cue in 50% of the trials. Odor delivery varied from 0.5 to 2-s to vary sensory uncertainty (Fig 1D). With the cue, mice responded faster to CS+ odors and rejected CS– odors more accurately in difficult trials (Fig 1E; RT: *t*(18) = –3.77, *p* = 0.001; error rate: *t*(18) = –4.02, *p* = 7.94 × 10⁻^4^). Combining accuracy and RT into the rate correct score (RCS) [30], we found that animals in cued trials maintained a high level of performance, which declined in un-cued trials (Fig 1F; 0.5 s odor window: *q*(36) = 6.37, *p* < 0.0001). The improvements in error and RT were most significant for 0.5 s odor duration. Interestingly, for events when odors were presented for the same duration as the training period (2 s), the auditory cue did not improve performance. This suggests that when odors provide sufficient information for decision-making, an added signal may interfere rather than enhance performance.

We sought to explore the neural basis of how attention modulates sensory representations to affect decisions. Since the 0.5 s foreperiod and odor delivery durations allowed the cue to provide the most striking change in performance, we used these parameters for further studies. Using widefield calcium imaging, we characterized odor-elicited glomerular responses in behaving mice carrying the *OMP-tTA; tetO-GCaMP2* alleles [31,32] (Fig 1G). We analyzed the glomerular activity across 19 animals performing this task. In un-cued trials, initial responses to CS+ and CS− odors were similar, but the responses quickly diverged as animals began licking waterspouts for CS+ odors (Fig 1H; [33]). Prior to odor delivery, the cue alone elicited glomerular responses before odor delivery (S1D and S1E Fig). Using ΔΔF/F to measure the differences between cued versus un-cued trials, we observed significant differences between cued versus un-cued CS+ responses in addition to overall CS+ versus CS− differences (Figs 1I–1K and S1F). On average, cued CS+ responses were 15% higher, with some glomeruli showing up to a 40% increase (cued versus un-cued CS+: *q*(18) = 4.05, *p* = 0.01). The cue strongly influenced the temporal dynamics of glomerular responses as response onset was significantly shorter for CS+, but longer for CS− odors (S1G Fig). Consistent with the behavioral impact of auditory cues based on the duration of odor, we did not observe cued enhancements for 2 s odor delivery (Figs 1L, left panel and S1H–S1L). We next quantified odor-selectivity of the glomeruli according to the difference between peak response amplitudes to the two odors (see “Materials and methods”). Cued versus un-cued CS+ responses were largely unchanged in glomeruli already selective for the same odor, but the difference increased in CS− selective glomeruli (Fig 1M, right panel). In contrast, response to cued CS− odor diminished at the CS− selective glomeruli. Taken together, these findings suggest that the cue selectively enhanced CS+ but suppressed CS− activity. We used a linear classifier to assess whether odor identity was accurately predicted based on the glomerular responses. This analysis revealed higher decoding accuracy during the odor period in the presence of cue, explaining the improvement of behavioral responses (Fig 1N; 0.5 s odor window: *q*(36) = 4.6, *p* = 0.0024). Selective modulation of odor responses was also observed in the postsynaptic mitral/tufted cell (MTC) dendrites expressing jGCaMP7f [34] (Figs 1O and S1M–S1Q). On average, cued CS+ responses at the MT dendrite were enhanced by 89%, nearly doubling the response compared to un-cued conditions (*q*(18) = 7.47, *p* < 0.0001). The cue also enhanced odor identity prediction from dendritic responses (Fig 1P; 0.5 s odor window: *q*(36) = 5.8, *p* = 0.0008). Thus, these results show that attention differentially modulates CS+ and CS− odor responses and improves odor identification.

### Cued-attention is mediated by a cholinergic HDB → SAC circuit

**Fig 2:**
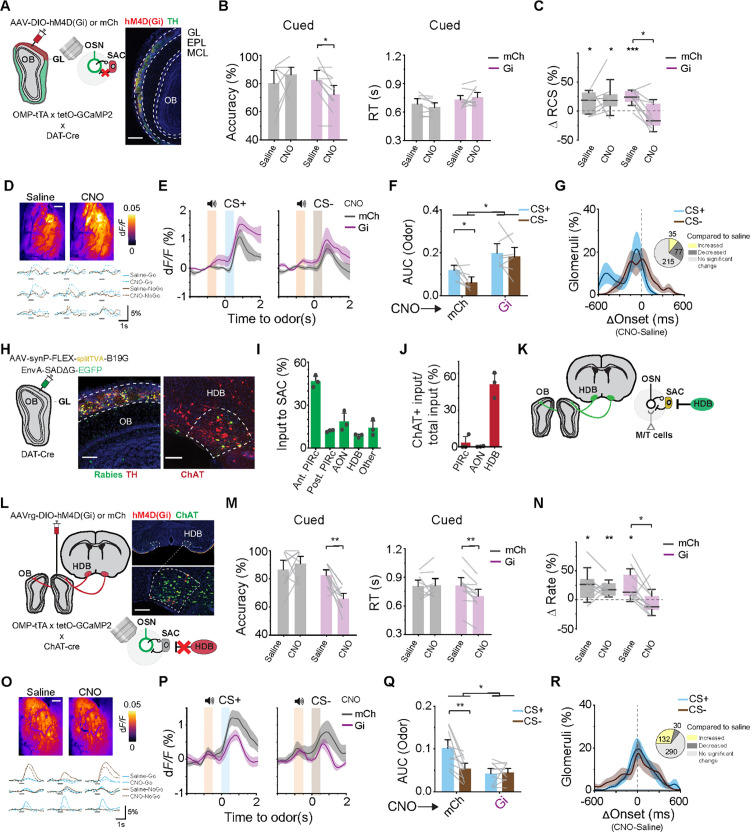
Cued-attention is mediated by a cholinergic HDB to SAC circuit. (A) Schematic of induced expression of DREADDi in SACs of *OMP-tTA; tetO-GCaMP2; DAT-Cre* animals and imaging of glomerular activity using GCaMP2. Example injection site shows Gi expression (red). Cellular layers in the OB are labeled as: GL: glomerular layers; EPL: external plexiform layer; MCL: mitral/tufted cell layer, scale bar: 200 µm. (B) Accuracy and RT differences between cued trials of CNO and saline injected (Gi; purple, *n* = 9) and mCherry (mCh; gray, *n* = 8) animals. *P < 0.05, mixed-design ANOVA followed by Tukey’s post hoc test. (C) RCS improvement of mCh and Gi mice after Saline or CNO injection. *P < 0.05, ***P < 0.001, one sample *t* test and mixed-design ANOVA followed by Tukey’s post hoc test. (D) Individual glomerular response across odor associated areas after saline and CNO injection in same animals, scale bar 200 µm. (E, F) Average traces (E) and bar graphs of AUC (F) showing glomerular responses following CNO injection in mCh or Gi expressing SACs. *P < 0.05, mixed-design ANOVA followed by Tukey’s post hoc test. (G) Distribution of differences in onset latency between CNO and saline injected trials for the same glomeruli. Inset denotes glomeruli with significant changes with SAC inhibition. Yellow: increased, dark grey: decreased, light grey: unchanged. (H) Left: Schematic of monosynaptic rabies tracing to identify inputs to SACs. Middle: signals in the OB. Yellow: starter cells for rabies virus. Right: dashed lines outline HDB areas. Green and yellow cells are retrogradely traced cells, scale bar: 100 µm. (I) Quantification of total input to SACs from different brain areas (*n* = 3). (J) Percentage of SAC projecting cells that are ChAT positive. (K) Proposed disinhibition circuit of the olfactory bulb mediated by cholinergic HDB. (L) Schematic of DREADDi expression in HDB cells projecting to OB of *OMP-tTA; tetO-GCaMP2; *ChAT-Cre** animals. Example injection site shows Gi expression (red) in cholinergic HDB (green), scale bar: 200 µm. (M, N) Same as (B, C) but for inhibition of HDB projections to OB. (Gi; purple, *n* = 10 or mCh; gray, *n* = 9 animals). **P < 0.01, mixed-design ANOVA followed by Tukey’s post hoc test. (O–R) Same as glomerular imaging in (D–G) but for inhibition of HDB projections to OB. *P < 0.05, **P < 0.01, mixed-design ANOVA followed by Tukey’s post hoc test, scale bar: 200 µm. Data for this figure are provided in S1 Data.

We hypothesized that changes in glomerular response arise from dis-inhibitory interactions in the OB. The short axon cells (SACs) innervate and inhibit multiple glomeruli via both GABA and dopamine [35]. OSNs express dopamine receptor D2, which is a GPCR coupled to Gi protein and facilitates inhibition [36]. The expression of tyrosine hydroxylase (TH), a key enzyme for dopamine synthesis, is regulated by sensory activity, making the SACs a likely target to regulate ongoing odor processing [37–39] ([33]). We tested whether cue-associated performance improvement required SACs. We crossed the *OMP-tTA; tetO-GCaMP2* mice with the dopamine transporter Cre (*DAT-Cre*) line and used AAV to express inhibitory DREADD receptors hM4D(Gi) [40] in the SACs (Figs 2A and S2A–S2C). Inhibition of SACs increased error in odor discrimination in cued trials (10.1% increase in error; *q*(15) = 4.74, *p* = 0.01), suggesting their role in integration of top–down signals to modulate bottom–up sensory responses (Fig 2B). DREADD-mediated inhibition of SACs did not significantly alter performance in trials without the auditory cue (S2D Fig). In mice transduced with the control AAV expressing mCherry, or in those transduced by hM4D(Gi) AAV but received saline injection, the cue improved RCSs. In contrast, there was no improvement when SAC activity was silenced by CNO injection (Fig 2C; Gi Saline versus CNO: *q*(15) = 4.46, *p* = 0.03).

To test whether these behavioral changes were accompanied by altered sensory encoding, we recorded OSN activity during chemogenetic manipulations. Suppression of SACs by CNO injection increased odor-evoked glomerular responses (Fig 2D–2F; *q*(12) = 4.88, *p* = 0.0048) and decreased onset latency in trained animals (Fig 2G). Decoding analysis of glomerular responses showed that under control conditions, the cue accelerated decoding, but SAC inhibition made cued and un-cued trials indistinguishable (S2E Fig). These results indicate that SACs are critical to fine-tune the sensory input and play an important role in cue-driven modulation of the responses. This conclusion aligns with previous studies proposing a modulatory role for SACs in shaping olfactory bulb activity and sensory gain [41–45].

We sought to identify the repertoire of direct input to the SACs, which may carry task-relevant information for early sensory modulation. By adopting monosynaptic tracing using pseudotyped rabies virus [46] (Fig 2H), we found that direct projection to SACs originated from known primary olfactory cortices, including the piriform cortex and the anterior olfactory nucleus (PIRc and AON, respectively; Figs 2I and S2F). Both have been proposed to mediate odor identity coding, but their role in attention-based enhancements of odor processing has not been reported [47,48]. The high number of cells may reflect the reciprocity between feedforward and feedback interactions. Approximately 8% of input to the SACs originates from the horizontal nucleus of the diagonal band (HDB), 50% of which were positive for choline-acetyltransferase (*ChAT*; Figs 2J and S3G). Despite the small number of neurons, the HDB has broad projection patterns and is implicated in olfactory and goal-directed behaviors [49–56]. It does not receive direct olfactory input, thereby likely provides context and state-dependent modulation of olfactory responses.

To determine whether the behavioral effects could be attributed to projections of basal forebrain input to the olfactory bulb, we performed a broad manipulation targeting all HDB → OB projections, regardless of neurotransmitter identity. Using a retrograde Cre-dependent DREADD strategy, we selectively silenced the entire basal forebrain projection population and assessed behavioral outcomes (S2H Fig). In pre-proficient mice, this manipulation abolished the cue-induced enhancement in performance (S2I–S2J Fig, *t*(12) = 3.15, *p* = 0.0083), indicating that basal forebrain input is necessary for attentional modulation in this task.

Muscarinic acetylcholine receptors are expressed in the glomeruli, and it is known that muscarinic activation disinhibits glomerular activity [28]. We, therefore, hypothesized that cholinergic HDB neurons provide an inhibitory signal to the SACs and attention utilizes this circuitry to modulate odor responses by reducing GABA and dopamine inhibition to the OSN axons (Fig 2K; [33]). To test this hypothesis, we crossed the *OMP-tTA; tetO-GCaMP2* mice with a *ChAT-Cre* line and bilaterally injected retrograde-AAV in the OB to express hM4D(Gi) in the long-range cholinergic projections from HDB to the OB (Figs 2L and S2K–S2L). Inhibition of cholinergic projections increased the error in odor discrimination (16.6% increase in error; Fig 2M; *q*(17) = 5.23, *p* = 0.008). We also noted a decrease in RT for the cued trials post CNO injection (111 ms decrease in RT; *q*(17) = 5.98, *p* = 0.002), suggesting that animals became more impulsive and less accurate after the manipulation. We found that the inhibition of cholinergic projections to the bulb did not alter performance in trials without the auditory cue in attention probe sessions (S2M Fig). Inhibition of *ChAT* positive inputs after training extinguished cue-induced performance enhancements (Fig 2N; Gi Saline versus CNO: *q*(17) = 6.46, *p* = 0.015). To further test whether HDB projections are required specifically during the attentional foreperiod, we optogenetically suppressed cholinergic axons in the OB using Archaerhodopsin during the cue–odor delay period (S2N Fig). This temporally precise manipulation abolished performance enhancement typically conferred in cued trials. These results, together with the SAC-targeted manipulations, support a model in which HDB cholinergic input enables attention modulation by transiently disinhibiting SACs. In contrast to SAC inhibition, which increased glomerular responses, suppression of cholinergic input to the OB reduced odor-evoked glomerular activity (Fig 2O–2Q; *q*(17) = 4.87, *p* = 0.0031). We also noted an increase in onset latency for odor evoked glomeruli activation regardless of odor-type (Fig 2R). Thus, cholinergic projection from the HDB is necessary for sensitization of early odor-evoked response and for cued-attention to improve behavioral performance.

### Cholinergic input modulates short axon cell responses

**Fig 3:**
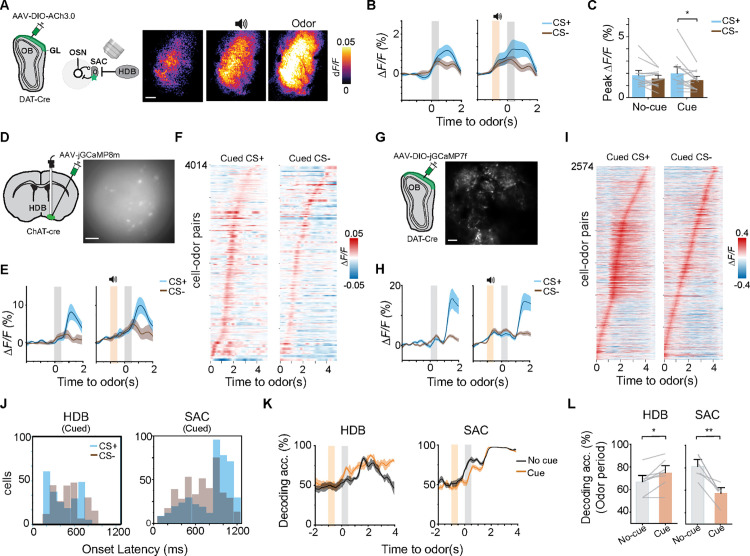
Cholinergic input modulates SAC responses. (A) Schematic for imaging ACh transients during cued-odor discrimination. Heatmaps show spatial distribution of ACh responses post cue and post odor, scale bar: 200 µm. (B) Average ACh transients for un-cued and cued trials (*n* = 13 animals). (C) Bar graph shows average peak responses. *P < 0.05, two-way repeated measures ANOVA followed by Tukey’s post hoc test. (D) Left: schematic of GRIN lens imaging of HDB neurons expressing GCaMP8m using a miniscope. Right: example imaging plane shows GCaMP expressing neurons, scale bar: 100 µm. (E) Population response traces show HDB neuronal activity from un-cued (left) and cued trials (right) in probe sessions (*n* = 8 animals). (F) Cell-odor pairs aligned to peak time for HDB activity. (G) Left: schematic of multiphoton imaging of SACs expressing GCaMP7f in *DAT-Cre* mice. Right: example of imaging plane showing SACs, scale bar: 50 µm. (H) Average SAC activity trace for un-cued (left) and cued odor stimulation (right; *n* = 6 animals). (I) Cell-odor pairs aligned to peak time for SAC activity. (J) Distribution of response onset latency of HDB cells (left) and SACs (right) in CS+ (blue) and CS− (brown) trials. (K, L) Linear decoding of stimulus identity in cued and un-cued trials from the HDB (left) and SAC (right) activities. *P < 0.05, **P < 0.01, one-way repeated measures ANOVA followed by Tukey’s post hoc test. Data for this figure are provided in S1 Data.

Our results suggest that cholinergic neurons modulate early odor responses through the dopaminergic SACs. The inhibition of these two sets of cells had opposite effects, consistent with an inhibitory role of the cholinergic input on the SACs. To directly visualize HDB cholinergic input to the SACs, we used AAV in *DAT-Cre* mice to express GRAB-ACh3.0, an acetylcholine (ACh) sensor [57] to record cholinergic transients (Fig 3A). In trained animals, cue-induced cholinergic activity was observed across the OB surface without apparent spatial bias associated with the glomerular activity evoked by odors [33]. The auditory cue induced a preparatory response similar to that seen in the glomerular responses. The valence of the stimuli determined the extent of odor-evoked cholinergic activity. The cue amplified differences between CS+ versus CS− odors (Fig 3B and 3C; *q*(12) = 4.29, *p* = 0.01). Thus, cholinergic activation triggered by the cue directly acts on SACs to enhance contrast between two task-relevant stimuli.

We sought to directly visualize HDB cholinergic cells to determine their role in the attention task. Using a miniscope, we imaged individual HDB *ChAT* neurons expressing jGCaMP8m [58] (Fig 3D; *n* = 235 cells from 8 animals). In trained animals, we observed a robust and sustained activation of these neurons exposed to CS+ odors, contrasting with a comparatively shorter duration and lower amplitude to CS− odors (Fig 3E–3F). Attentional cue elicited a distinctive ramping activity prior to odor-elicited response, suggesting an increased responsiveness to the impending olfactory stimulus. Notably, the activity ramp was indistinguishable between CS+ and CS− odors (S3A Fig). The difference between post-odor cued and un-cued responses revealed further enhancement of CS+ odor response (S3B Fig). These findings indicate a dual role for HDB *ChAT* neurons in both attentional modulation and reward-based enhancement.

Since the SACs inhibit the OSN axons, inhibitory input from the HDB to the SACs would disinhibit the OSN input, which in turn activate SACs through glutamatergic activation. We monitored calcium signals from the SAC cell bodies using multi-photon imaging in *DAT-Cre* mice transduced with AAVs that conditionally express jGCaMP7f (Fig 3G; *n* = 358 cells from 6 animals). The attentional cue engaged SACs, eliciting an increase in calcium signals that was followed by response to odor delivery and to reward (S3C Fig). Since the only known excitatory input to the SACs is from the OSNs, we interpret the cue-induced signal as elevated basal activity from disinhibition of the OSNs. While the cue-induced responses were similar in amplitude for both CS+ and CS− trials, odor elicited responses differed depending on valence (Figs 3H–3I and S3D). We observed a shorter onset latency for odors in HDB neurons compared to SACs (Fig 3J). In SACs, CS+ response was delayed compared to CS− odor, likely resulting from a stronger HDB inhibition. Using linear decoding of HDB response we observed higher accuracy in decoding the stimuli when trials were cued (Fig 3K and 3L; HDB: *q*(7) = 4.88, *p* = 0.01; SAC: *q*(5) = 8.39, *p* = 0.0019). In contrast, the SAC activity showed a lower prediction accuracy during stimulus presentation in cued trials, indicating an inhibitory drive to the SACs delays the response. This is consistent with the model that HDB inhibits SACs to enhance early odor detection during attention engagement, thereby facilitating rapid processing of CS+ odors.

### Dynamic engagement of attention under varying cognitive demand

**Fig 4:**
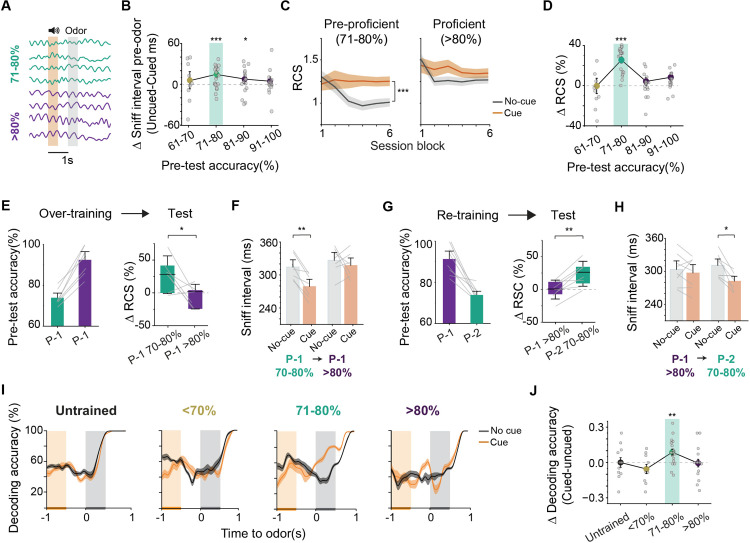
Dynamic engagement of attention under varying cognitive demand. (A) Breathing traces from one animal across two levels of training accuracy: 71%–80% (olive) and >80% (purple). (B) Difference in sniff intervals with and without the cue prior to odor delivery in animals at different pre-test performance accuracy. 61%–70% (*n* = 9 animals), 71%–80% (*n* = 19 animals), 81%–90% (*n* = 16 animals), 91%–100% (*n* = 14 animals). Olive color shade highlights the pre-proficient accuracy level (71%–80%). Dashed line represents no difference between trials. *P < 0.05, ***P < 0.001, one sample *t* test. (C) RCS for cued and un-cued trials for pre-proficient (left panel) and proficient (right panel) animals across the entire session. ***P < 0.01, one-way repeated measures ANOVA followed by Tukey’s post hoc test. (D) Improvement of RCS by cue in test sessions segregated according to pre-test performance accuracy. ***P < 0.001, one sample *t* test. (E) Left: Odor discrimination accuracy as pre-proficient animals transition to proficient levels following training on the same odor pair. Right: Cue-based improvements in the same animals before and after additional training (*n* = 7 animals). *P < 0.05, one-way repeated measures ANOVA followed by Tukey’s post hoc test. (F) Sniff intervals before and after additional training. **P < 0.01, two-way repeated measures ANOVA followed by Tukey’s post hoc test. (G) Left: Performance accuracy for proficient animals trained on a second odor pair (P2) to pre-proficient level. Right: Cue-based improvement of RCS for proficient animals before and after retraining with a new odor pair to pre-proficient level (*n* = 8 animals). **P < 0.01, one-way repeated measures ANOVA followed by Tukey’s post hoc test. (H) Sniff intervals before and after re-training. *P < 0.05, two-way repeated ANOVA followed by Tukey’s post hoc test. (I) Decoding accuracy based on glomerular responses using SVM across cued vs. un-cued trials in the same session for mice at different pre-test proficiency levels or naïve (*n* = 11 animals). (J) Difference in decoding accuracy between cued and un-cued trials for individual mice in (B) and (D). Olive-color shading highlights data from the pre-proficient group. **P < 0.01, one sample *t* test. Data for this figure are provided in S1 Data.

Attention requires a heightened alert state. Odor stimulation triggers faster sniffing and animals can decode information conveyed by sniff cycles and use it to navigate odor gradient [59–61]. In trained mice, we observed faster preparatory sniffing prior to odor delivery in cued but not un-cued trials (Fig 4A). We further divided the mice according to their behavioral performances. Taking variability into account, we considered mice performing below 60% as at chance, whereas those above 80% as proficient [62]. We further divided mice performing between 60% and 80% into novice (61%–70%) as they were still grappling with the behavioral paradigm, and pre-proficient (71%–80%) for those already mastered the task but have not reached criterion. The pre-proficient mice engaged in faster breathing than the novice or the proficient mice (Figs 4B and S4A–S4E; pre-proficient: *t*(18) = 4.21, *p* = 5.84 × 10⁻^4^; proficient (81%–90%): *t*(15) = 2.77, *p* = 0.01). The cue did not change odor sampling after odor delivery (S4F–S4K Fig). These observations indicate that the cue increased arousal at low but not high proficiency levels, revealing an inverse relationship between arousal and task performance.

We tested how attention affects performance at different proficiency levels. In pre-proficient animals, performance remained stable in cued trials but dropped without the cue. This showed that attention helps maintain learned discrimination (Fig 4C; pre-proficient: effect of trial type *q*(18) = 11.83, *p* < 0.0001; proficient: *q*(32) = 2.59, *p* = 0.076). Behavioral improvement was most pronounced for pre-proficient animals (Figs 4D and S4L–S4N; pre-proficient: *t*(18) = 8.59, *p* < 0.0001). Indeed, when pre-proficien*t* mice reached proficiency, the cue no longer improved RCS, nor did it engage faster breathing (Fig 4E and 4F; RCS: *q*(6) = 3.76, *p* = 0.037; sniff interval: *q*(6) = 4.27, *p* = 0.023). Moreover, when we trained animals that were proficient in discriminating one pair of odors to pre-proficient levels for a new pair of odors, the cue reinstated pre-odor arousal and improved RCS (Fig 4G and 4H; RCS: *q*(7) = 6.4, *p* = 0.002; sniff interval: *q*(7) = 4.02, *p* = 0.024). The contingency switch results demonstrate not only the dynamic nature of attention but also its utility under high cognitive demand, where task difficulty requires additional cognitive effort to maintain accuracy and speed.

To understand if dynamicity in attention allocation is reflected in the sensory responses, we analyzed the glomerular activity across pre-test groups and used linear decoding to assess whether odor identity was accurately predicted. This analysis revealed higher decoding in presence of cue only in the pre-proficient mice (Fig 4I and 4J; *t*(18) = 3.43, *p* = 0.003). We also noted a subdued attention-guided selection for CS+ odors in proficient animals (S5A–S5D Fig). These results highlight the adaptive nature of attentional modulation, revealing that attention enhances sensory processing and decision-making mainly during intermediate learning stages, when task difficulty and arousal are balanced.

### Engagement of cholinergic basal forebrain underlies differential attention effect

**Fig 5:**
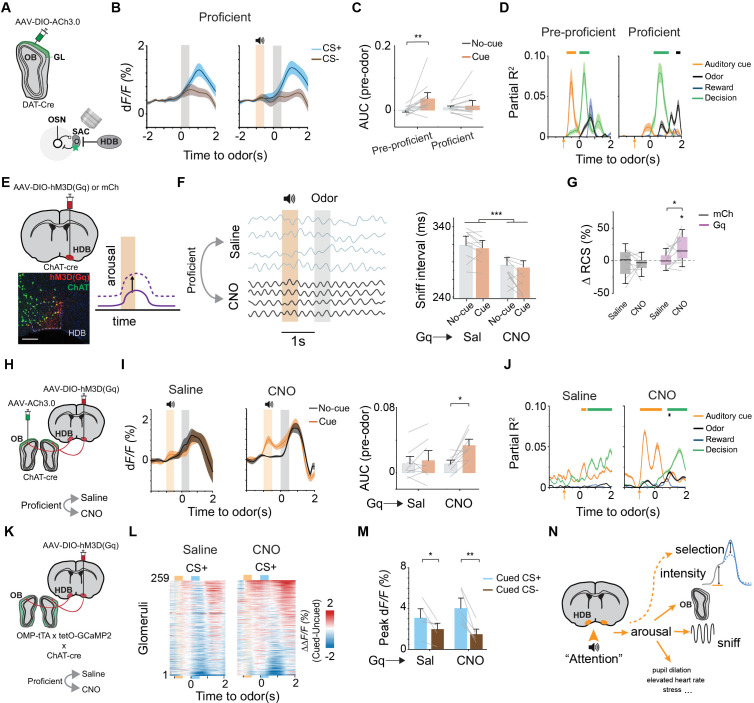
Engagement of cholinergic basal forebrain underlies differential attention effect. (A) Schematic for imaging ACh transients during cued-odor discrimination across proficiency level. (B) Average ACh transients for un-cued and cued trials at proficient levels (*n* = 13 animals). (C) AUC measurement of responses for pre-odor period (*t* = −1 to 0 s). **P < 0.01, two-way repeated measures ANOVA followed by Tukey’s post hoc test. (D) Linear model with stimulus and decision predictors for ACh activity (See “Materials and methods”). Solid lines on top indicate **P* *< 0.01. (E) Schematics of DREADDq (Gq; purple, *n* = 10 animals) or mCherry (mCh; gray, *n* = 8 animals) expression in cholinergic HDB of *ChAT-Cre* animals. Example injection site shows Gq expression (red) in cholinergic cells (green). Scale bar: 200 µm. Chemogenetic activation of HDB was expected to increase arousal in proficient mice (right panel). (F) Breathing traces (left) and bar plot (right) showing sniffing intervals from a proficient mouse before (Saline) and after (CNO) HDB activation. ***P < 0.001, two-way repeated measures ANOVA followed by Tukey’s post hoc test. (G) RCS improvement of mCh and Gq mice after saline or CNO injection in proficient animals. *P < 0.05, one sample *t* test and mixed-design ANOVA followed by Tukey’s post hoc test. (H) Schematic for imaging ACh transients in proficient *ChAT-cre* mice expressing Gq in HDB. (I) ACh transients (left) and AUC measurements for pre-odor period (right) for un-cued and cued trials (*n* = 9 animals) before (Saline) and after (CNO) HDB activation. *P < 0.05, mixed-design ANOVA followed by Tukey’s post hoc test. (J) Same as (D) but for ACh release contingent on HDB activation. (K) Schematic for imaging glomerular responses in proficient mice expressing Gq in HDB. (L) Heatmaps show the difference of activity between cued and un-cued trials (*ΔΔF*/*F*) for the same glomeruli in proficient mice before (Saline) and after (CNO) HDB activation. (M) Bar graph shows average peak responses. *P < 0.05, **P < 0.01, mixed-design ANOVA followed by Tukey’s post hoc test. (N) Schematic showing that HDB activation may increase arousal, which in turn modulates multiple physiological processes and may directly or indirectly affect the intensity and selectivity of sensory responses. Data for this figure are provided in S1 Data.

The differential effect of cued-attention on behavioral performance may arise when local circuits cease to respond to attention signal from the central brain. Alternatively, the engagement of cholinergic neurons dictates the differential effect. To distinguish the two possibilities, we recorded cholinergic activity as animals transitioned from pre-proficiency to proficiency (Fig 5A). In pre-proficient mice, ACh transients were reliably activated by the cue before odor delivery (*q*(12) = 4.35, *p* = 0.009), but when the same mice became proficient the cue no longer activated ACh transient even though odor exposure did (Fig 5B and 5C; *q*(12) = 0.63, *p* = 0.66). We created a linear model to predict ACh activity using stimuli and decision variables. This model revealed how each factor uniquely influenced ACh signals over time. Auditory stimulus was the largest contributor to the variance in ACh activity before odor was presented (Fig 5D). This contribution was noticeably lower for proficient animals, indicating a reduced influence of the auditory stimulus on ACh release.

In contrast to the cue, odor elicited ACh transient was reward dependent regardless of proficiency level. Stronger transients were elicited by the rewarded odor than the penalty odor. At pre-proficient levels, the cue amplified differences between CS+ versus CS− odors (S5E and S5F Fig). At high levels of proficiency, the contrast between CS+ and CS− responses were both prominent with and without the cue (S5G Fig). We reasoned that the differential ACh signals might influence the behavioral outcome. In this case, ACh release was expected to correlate with decisions rather than odors; thus, we segregated ACh activity data according to the decision responses. ACh transients were prominent in both Hit and False Alarm decisions, when mice licked the waterspout, but they were subdued in Miss and Correct Rejection decisions (S5H Fig). To determine whether odor evoked ACh transients reflect motor output or reward expectation, we compared responses between Hit and False Alarm trials. Both involve motor actions but only Hits are followed by a reward. In both cued and un-cued trials, ACh transients were significantly higher in Hit trials compared to FAs (S5I Fig), suggesting that cholinergic responses are not merely driven by the presence of a motor act. Instead, they also reflect reward expectation or receipt. To further assess this, we fit logistic regression models to predict the probability of a Hit based on odor AUC (S5J Fig). The model revealed that higher AUC values corresponded to a higher probability of Hit responses, and this relationship was enhanced under cued conditions (Likelihood ratio test (LRT) statistic: 6.96, *p* = 0.008; Un-cued versus null: LRT Statistic: 2.81, *p* = 0.09). Together, these findings indicate that ACh activity is associated with both reward and motor initiation. ACh-mediated disinhibition promotes reward seeking response.

Since cholinergic HDB was disengaged in proficient animals, we reasoned that re-engaging it will increase arousal. To test this, we bilaterally expressed hM3D(Gq) in the HDB and trained the animals to proficiency. We injected CNO or saline prior to odor discrimination trials (Fig 5E). CNO, but not saline, led to a significant increase in tonic arousal as observed from their sniffing bouts (Figs 5F and S5K; *q*(9) = 12.93, *p* < 0.0001). In proficient animals, while the cue did not cause additional arousal, activation of the *ChAT* neurons was sufficient to enhance auditory cue-associated odor discrimination (Fig 5G; *q*(16) = 4.3, *p* = 0.034). We then recorded ACh transients from the OB and found that CNO increased cue-induced cholinergic activity (Fig 5H–5J; *q*(8) = 4.32, *p* = 0.015). These results indicate that cholinergic HDB cells regulate attentional intensity to enhance discrimination.

As cholinergic basal forebrain is implicated in regulating arousal for general wakefulness, novelty seeking, and cue-valence association, we used a novelty seeking paradigm to probe odor investigation behaviors [63] (S6A Fig). CNO activation of cholinergic cells in HDB led to substantial increase in odor investigation compared to saline (S6B Fig). Control animals injected with CNO, or saline, exhibited similar levels of odor investigation over air, as measured by the investigation index (S6C Fig). Interestingly, cholinergic enhancement also drove investigation of the odor port between odor sessions when no odor was present (S6D Fig). This took place after the animals were exposed to the first odor delivery, suggesting a persistent odor-seeking behavior. This indicates that cholinergic neuron engagement is sufficient to increase arousal and odor driven investigation.

To evaluate modulation of sensory response, we recorded calcium signals from glomeruli of *OMP-tTA; tetO-GCaMP2; ChAT-Cre* mice (Fig 5K). In proficient animals, valence dependent bias was predominant. The auditory cue did not increase the activity for CS+ odor over un-cued trials. Chemogenetic activation of cholinergic activity was sufficient to increase CS+ responses in the presence of cue (Fig 5L). Moreover, the activation of cholinergic modulation selectively increased glomerular responses, accentuating the contrast between CS+ and CS− odors (Fig 5M; Saline: *q*(5) = 3.83, *p* = 0.042; CNO: *q*(5) = 5.83, *p* = 0.009). Taken together, these results demonstrate that the increase in cholinergic signal drives attention. In proficient animals, where natural cue-evoked cholinergic transients are diminished, reactivation of this pathway reinstates cue sensitivity and sharpens odor contrast, underscoring the circuit’s role in adaptive attention. However, it is not clear whether attentional intensity during the pre-odor period and selective odor response are the result of general arousal, which serves functions such as regulating breathing, pupil dilation, and stress, or from the specific action on sensory areas (Fig 5N).

### Reinstating attentional effects in the absence of global arousal

**Fig 6:**
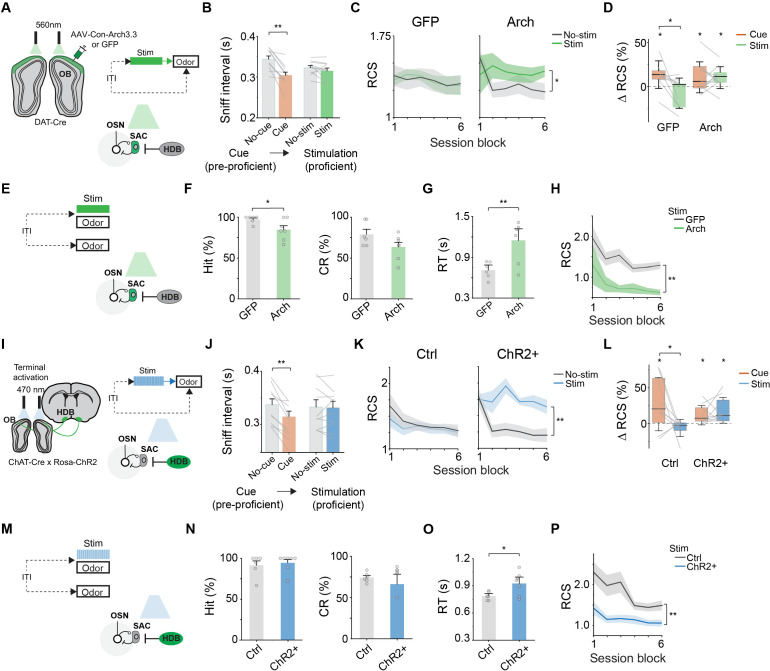
Reinstating attentional effects in the absence of global arousal. (A) Strategy for optogenetic inhibition of SACs. *DAT-Cre* animals that were injected with Archaerhodopsin (Arch) or GFP. LED stimulation from *t* = −1 to 0 s, odor is delivered from *t* = 0 to 0.5 s. (B) Comparison of average sniff intervals of pre-proficient animals in un-cued (gray) vs. cue trials (orange) and of proficient animals without (gray) and with (green) pre-odor light stimulation to dampen SAC activity. **P < 0.01, two-way repeated measures ANOVA followed by Tukey’s *post hoc* test. (C) RCS for stimulated and un-stimulated trials of GFP (left panel, *n* = 9) and Arch injected (right panel, *n* = 11) proficient animals across the entire session. *P < 0.05, one-way repeated measures ANOVA followed by Tukey’s *post hoc* test. (D) Improvement in RCS for GFP and Arch mice after cue presentation (pre-proficient) or pre-odor stimulation (proficient). *P < 0.05, one sample *t* test and mixed-design ANOVA followed by Tukey’s *post hoc* test. (E) Strategy for optogenetic inhibition of SACs concurrently with odor stimulation. (F) Comparison of hit rate and correct rejection between stimulated trials of GFP (*n* = 6) and Arch (*n* = 8) injected proficient animals for the same odors. *P < 0.05, Two sample *t* test (two-tailed) (G) Comparison of RT for CS+ odor between stimulated trials of GFP and Arch injected animals. **P < 0.01, Two sample *t* test (two-tailed). (H) RCS for odor concurrent stimulation trials of GFP and Arch proficient animals across the entire session. **P < 0.01, Two sample *t* test (two-tailed). (I) Strategy for optogenetic excitation of cholinergic projections in the OB in *ChAT-cre; R-ChR2* (ChR2+) and *ChAT-cre* (Ctrl) animals. Top: 50 Hz LED stimulation from *t* = −1 to 0 s, odor is delivered from *t* = 0 to 0.5 s. (J–L) Same as (B–D) but for excitation of cholinergic projections pre-odor delivery (*n* = 10 animals for Ctrl and ChR2+ conditions each). (M–P) Same as (E–H) but for excitation of cholinergic projections concurrently with odor delivery (*n* = 6 animals for Ctrl and *n* = 7 animals ChR2+ condition). Data for this figure are provided in S1 Data.

We sought to determine whether the effect relied on global arousal, or it could be replicated by selectively activating the bulbar circuit without global arousal. We optogenetically manipulated the activity of SACs and cholinergic input in proficient mice with high temporal resolution. We directly inhibited SACs after bilateral expression of archaerhodopsin (Arch3.3) [64] in the *DAT-Cre* mice (Fig 6A). Light stimulation just prior to odor delivery did not enhance preparatory sniffing but maintained high RCS across the session (Fig 6B and 6C; sniff interval: *q*(10) = 1.11, *p* = 0.45). GFP-injected pre-proficient mice showed improved task performance with the auditory cue but not with optical stimulation when they became proficient. Brief pre-odor inhibition of SACs increased performance over non-stimulated trials at a level comparable to cued-attention (Fig 6D; Cue versus Stim in Arch injected animals: *q*(18) = 0.07, *p* = 0.99).

We also investigated the timing of SAC inhibition on behavior responses in proficient animals (Fig 6E). Light inhibition of SACs concurrent with CS+ odor presentation decreased Hit decisions and did not increase behavioral response to the CS− odor above control animals, although there was a trend toward reduced correct rejection decisions compared to control animals (Fig 6F; Hit rate: *t*(11) = 2.28, *p* = 0.043; CR rate: *t*(11) = 1.91, *p* = 0.081). SAC inhibition increased RT in Arch injected animals (Fig 6G; *t*(11) = –3.24, *p* = 0.0078). A comparison of stimulated odor trials revealed an overall decrease in performance when SACs were inhibited concurrently with odor presentation (Fig 6H). This result indicates that only the preparatory inhibition of SACs improves performance. Concurrent light stimulation with odor presentation impairs performance, possibly because it indiscriminately disinhibits odor responses and reduces odor discrimination.

We next used optogenetics to stimulate bulb-projecting cholinergic fibers in proficient *ChAT-Cre; R-ChR2* mice (Fig 6I). In channelrhodopsin (ChR2) [65] expressing mice, light activation just prior to odor delivery improved accuracy without triggering preparatory sniffing (Fig 6J and 6K; sniff interval: *q*(9) = 0.45, *p* = 0.75). The improvement was comparable to that seen with cued-attention in pre-proficient ChR2 expressing mice (Fig 6L; Cue versus Stim in ChR2 animals: *q*(18) = 0.84, *p* = 0.55). This mimicked the effect of the attention cue, suggesting that direct cholinergic activation in the olfactory bulb can enhance behavior without global arousal. We next examined whether cholinergic stimulation during odor presentation would similarly improve or behavioral outcomes (Fig 6M). In proficient animals, light delivered concurrently with CS+ odor presentation had no effect on the hit rate and correct rejection decisions (Fig 6N; Hit rate: *t*(11) = −0.45, *p* = 0.65; CR rate: *t*(11) = 0.6, *p* = 0.55). Stimulation of cholinergic fibers increased RT (Fig 6O; *t*(11) = –2.53, *p* = 0.027). This suggests that direct recruitment of cholinergic activity in the OB does not increase impulsivity in decision-making. Across session, cholinergic stimulation resulted in low performance compared to stimulated controls (Fig 6P). These results reinforce the notion that preparatory disinhibition, rather than disinhibition *per se*, was responsible for performance enhancement.

### Modeling plasticity dependent attention selectivity and adaptation

**Fig 7:**
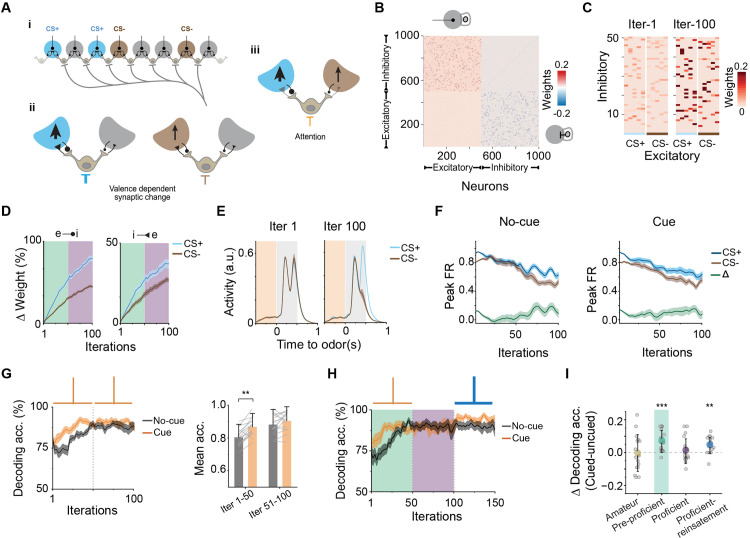
Modeling plasticity dependent selective attention. (A) Schematic of the glomerular network model incorporating leaky-integrate-and-fire (LIF) neurons to simulate odor-driven activity and cholinergic modulation in the olfactory bulb. (i) Diagram showing selective activation of a subset of excitatory olfactory sensory neurons (OSNs, circles) by conditioned stimuli (CS+ or CS−). OSNs form reciprocal connections with inhibitory short axon cells (SACs, small circles with connections). CS+ trials (blue) selectively activate a specific OSN-SAC ensemble, while CS− trials (brown) activate a different subset. Network wiring enables targeted modulation of specific glomeruli based on stimulus identity. (ii) Valence-dependent synaptic changes within the OSN-SAC microcircuit. Differential cholinergic input (blue arrow for CS+, brown arrow for CS−) modulates SAC inhibition, shaping OSN-SAC connectivity via a Hebbian learning rule. Increased inhibition of SACs during CS+ trials promotes disinhibition of OSNs, enhancing their activity, while reduced inhibition during CS− trials maintains higher SAC-mediated suppression, thereby reducing bottom–up activation of SACs. (iii) Global cholinergic input simulating attention exerts uniform inhibition onto SACs across glomeruli. The functional impact is shaped by differential connectivity: SACs which have stronger inhibitory connections to OSNs, are more strongly suppressed by the same cholinergic signal, effectively biasing glomerular responses during attentive states. (B) Random initial connectivity matrix of excitatory and inhibitory clusters. The 1st quadrant represents excitatory weights from putative glomeruli to SACs; the 4th quadrant represents reciprocal inhibitory weights from SACs to glomeruli. (C) Changes in synaptic weights after training for CS+ and CS− stimuli. (D) Cumulative update in weights for the 2 stimuli. Olive shading indicates training up to “pre-proficient” level and purple indicates “proficient” level remodeling of synaptic strengths. (E) Average activity of excitatory clusters with input and attention signals pre- and post- valence dependent remodeling. (F) Peak activity changes across learning. Green represents the difference in peak responses for CS+ and CS−. (G) Decoding accuracy of stimulus identity with or without the attention cue across learning. Attention signal was kept at the same gain throughout iterations. Right: Average decoding accuracy across remodeling stages. ***P < 0.001, two-way repeated measures ANOVA followed by Tukey’s post hoc test. (H) Decoding accuracy of stimulus identity with or without the attention cue across learning. Blue sign indicates recapitulation of attention by reengagement of the attention signal post proficiency. (I) Difference in decoding accuracy between cued and un-cued simulations for 16 pairs of input patterns using the remodel. **P < 0.01, ***P < 0.001, one sample *t* test. Data for this figure are provided in S1 Data.

To obtain a theoretical understanding of the function of this circuit, we simulated neuronal interactions with a network model composed of leaky integrate-and-fire neurons representing OSNs and SACs, which form reciprocal excitatory and inhibitory synapses (Fig 7A). Connectivity between the inhibitory and excitatory clusters mimicked the interaction between OSN axons and the SACs (Fig 7B). ACh input was modeled as a negative bias to the baseline current of SACs. Our experimental data demonstrated that ACh release is triggered by the attentional cue, which does not distinguish between CS+ and CS− stimuli, and by post-odor activity, which shows a significant difference depending on odor valence. We reason that the two distinct phases of ACh activity have differential effects on odor discrimination. To test this, we modeled odor-dependent disinhibitory drive by providing a strong ACh bias associated with the CS+ but a weak one with the CS− odor. The differential top–down inhibition did not affect activity in inhibitory and excitatory neurons (S7A Fig), indicating that cholinergic modulation alone is insufficient to drive robust stimulus separation in a naïve circuit. We then introduced a non-selective pre-odor ACh input, mimicking the cue-triggered attentional foreperiod observed in our physiological data. This led to increased firing rates in both inhibitory and excitatory neurons, consistent with calcium imaging results showing elevated pre-odor activity. However, SVM decoding showed no improvement in odor classification with the addition of the pre-stimulus input (S7B Fig).

How does a global disinhibitory input enable differential modulation of sensory input? Spike-timing-dependent plasticity facilitates Hebbian learning and has been proposed to enhance pattern separation in the olfactory bulb [66–68]. Our companion study indicated valence dependent plasticity in the OB circuit. We hypothesized that these changes underly the attentional effect. Strong ACh release associated with CS+ odors would provide a strong disinhibition of the glomeruli and strengthen the reciprocal synapses between OSNs and SACs. Consequentially, when an attention-driven cholinergic signal arrives in the OB, inhibition of SACs with stronger synaptic connections preferentially disinhibits CS+ but not CS− OSN axons, leading to differential responses. To test this hypothesis, we allowed the initial random weights to undergo Hebbian learning. Enhanced activity in excitatory clusters resulted in strengthened connectivity with their corresponding inhibitory clusters over multiple iterations (Figs 7C and S7C). Moreover, the increase in connectivity was correlated with disinhibitory strength (Fig 7D). Training biased CS+ responses in the excitatory clusters (Fig 7E). At various stages of training, we simulated the attentional effect with pre-stimulus disinhibition. This effectively lowered the threshold for excitatory neuron activation in response to subsequent odor cues and consistently enhanced the contrast between CS+ and CS− activity across the training period (Fig 7F). Linear classification indicated that pre-stimulus bias significantly improved discrimination over time (Fig 7G; *q*(15) = 4.95, *p* = 0.0032). Notably, the effect of attentional cue decreased as selective strengthening of synapses progressed (*q*(15) = 1.81, *p* = 0.21), underscoring the adaptation of attention to task demands. However, at the “proficient” level, a stronger pre-stimulus disinhibition can further improve discrimination, recapitulating our experimental findings (Fig 7H and 7I, pre-proficient: *t*(15) = 4.31, *p* = 6.18 × 10⁻^4^; reinstatement: *t*(15) = 3.78, *p* = 0.0018).

## Discussion

Attention is a dynamic cognitive process that enables animals to prioritize sensory inputs based on goals, expectations, and past experience. Recapitulating attention in experimental settings has proven challenging, due to the absence of a clear understanding of the circuit and the molecular mechanisms that govern its regulation. Basal forebrain is one of the three major cholinergic systems in the brain [69]. HDB projects across memory systems in the hippocampus, prelimbic area, olfactory associated regions, and the primary sensory cortices [70,71]. Cholinergic signaling has been found necessary for goal-directed behaviors and is implicated in many cognitive functions, including arousal, memory, and reward [72–74]. Although cholinergic projections from the basal forebrain have been implicated in modulating attention across sensory and associative cortices, the precise mechanisms and circuit-level specificity have remained elusive. In the olfactory bulb, cholinergic signaling has been associated with enhanced odor detection, discrimination, and sensory gain [49–56]. However, these effects have largely been attributed to broad modulations with limited insight into how cholinergic input could create stimulus-specific enhancements at early stages of sensory processing.

Here, we identify a disinhibitory circuit motif, the HDB → SAC → OSN pathway that enables selective enhancement of task-relevant sensory inputs. In pre-proficient animals, cue presentation triggers cholinergic release prior to odor onset. This preparatory phase transiently suppresses SAC activity, disinhibiting OSN terminals, and increasing their basal calcium levels. During odor presentation, glomerular responses are further shaped by odor valence: CS+ odors evoke stronger cholinergic transients, leading to enhanced OSN activation through sustained SAC suppression. By identifying reciprocal OSN ↔ SAC interaction as the circuit motif that enables modulation of sensory input, we demonstrate that even these earliest processing stages are subject to such attention-based modulation. Attention increases the speed and amplitude of glomerular responses, which supports faster, more accurate decisions and helps animals avoid errors. Although the response amplitude to penalty odors did not change, glomeruli became more selective for CS+ odors. As glomerular activity controls synaptic transmission to postsynaptic neurons, this presynaptic modulation not only gates the information transfer to relay neurons but is essential for improving decision-making.

We show that cue-induced cholinergic activity does not distinguish between the odors as observed in the spatial patterns, nor in amplitude associated with odor valence. Thus, cued-attention is a broadcast signal sent to all glomeruli in the OB. On the other hand, odor-driven cholinergic activity differentially modulates odor activation patterns. Our experimental and modeling studies show that experience-dependent mechanisms can drive odor-specific response. Sustained ACh activity contingent on reward status creates an opportunity to generate selective changes in synaptic strength associated with individual odors. Prolonging cholinergic input into the SACs prolongs disinhibition of the glomeruli. Pairing between glomerular and SAC activities enhances reciprocal synaptic interactions for reward odors in our network model. The penalty odor does not produce the same prolonged SAC activity. Thus, cholinergic inputs not only provide transient modulation but also initiate long-term plasticity that enhances sensory discrimination. Importantly, discriminative learning induces structural and molecular changes in SACs, particularly an increase in TH expression around glomeruli associated with CS+ odors [33]. Furthermore, this molecular plasticity supports the computational model proposed in the present study, where synaptic strengthening during learning reduces reliance on attention over time. While previous models have demonstrated that cholinergic inputs can affect olfactory bulb transformations [75,76], these implementations primarily explored concomitant neuromodulatory gain effects. In contrast, our computational model incorporates a dynamic interaction between top–down cholinergic input and learning-modulated SAC inhibition, enabling selective amplification of CS+ odors during cued-attention. The model captures how behavioral performance in pre-proficient animals depends on the presence of the cue, and how glomerular responses change over the course of training.

We find that attention has the largest effect on performance when animals begin to distinguish the odors to obtain reward and avoid punishment. The cue does not inherently enhance performance but helps maintain learned performance levels. As animals become proficient at odor discrimination, the effectiveness of auditory cues in improving performance diminishes. The adaptive changes thus result in an inverted U-shape curve with a Goldilocks zone where attention is allocated to improve performance. This suggests that cholinergic input from HDB acts as a dynamic, experience-dependent attentional signal. During early learning, cue-triggered ACh enhances CS+ responses via SAC disinhibition, facilitating faster and more accurate decisions. However, as task proficiency increases and odor-reward associations become stabilized in the circuit, this top–down modulation disengages. Mice no longer rely on external attentional cues but instead utilize locally stored sensory templates shaped by prior learning to perform the routine task. Importantly, this disengagement does not reflect a loss of cholinergic tone. The HDB cholinergic neurons respond robustly to odor stimulation and reward. Moreover, they can be quickly engaged when odor contingency is shifted, when new pairs of odors need to be distinguished. The inverted U-shape curve here is reminiscent of, but distinct from, the Yerkes–Dodson law—where performance increases with mental arousal but declines when arousal becomes excessive [77]. The relationship we find here is specific to attention and the result of disengagement of the cholinergic system. Performance does not decline, only that the effect of attention is decreased. Nevertheless, the resemblance between these two curves raises the question about the relationship between attention and arousal. Arousal represents a heightened state of the brain. It is a precondition for attention but does not necessarily improve performance as the Yerkes–Dodson curve indicates. It is possible that an elevated level of arousal may engage too many cognitive processes and result in an overall compromise in performance. Our experiments show that when an odor is presented for 2 s, cued-attention in fact slightly reduces odor discrimination, indicating that when sensory information is sufficiently available for decision-making, an additional signal interferes instead of improves performance [78]. Thus, the disengagement of cholinergic neurons in proficient animals serves as a mechanism to remove unwanted signals and reduce interference. It is worth noting that re-engagement of the OB specific cholinergic circuit can still improve performance. Thus, the effect of attention is separable from global arousal.

Recent evidence reveals the complexity of cholinergic modulation in the olfactory bulb [79,80]. Cholinergic modulation in the olfactory bulb involves both nicotinic and muscarinic receptors. Nicotinic receptors are present, particularly on MTCs and GABAergic interneurons [80]. The excitatory effect of nicotinic receptors contributes to shaping postsynaptic MTC responses but is less consistent with the inhibitory effect on SACs [79]. Our findings are more consistent with muscarinic receptor-mediated inhibition of SACs [28]. This inhibitory modulation reduces SAC output, thereby disinhibiting OSN terminals and enhancing early glomerular responses. However, we cannot exclude additional contributions of nicotinic signaling to the attentional process, particularly in postsynaptic neurons. Within the glomerular layer, the SACs and the periglomerular cells (PGCs) express muscarinic receptors and form reciprocal inhibitory synapse to create an excitation-inhibition balance [81]. Both the HDB → PGC and the PGC → SAC pathways are inhibitory [28,81]. HDB activation will result in an increased glomerular response from inhibition of PGC, but it will likely enhance SAC-mediated inhibition of glomerular activity by inhibiting the PGC → SAC pathway. Together these effects may be counterbalanced at the same glomerulus. Unlike the SACs, which extend inter-glomerular connections, the PGCs release GABA onto the OSN terminals within a single glomerulus [81,82]. A broad, non-specific cholinergic signal is expected to inhibit the PGCs similarly across the glomeruli, which may not contribute to specific enhancement of glomerular activity. The inter-glomerular projection of the SACs allows lateral inhibition to create selective enhancement of glomerular responses and contrast between odor-evoked patterns. Co-transmitting HDB projection also excites deep SACs (dSACs) in the inner plexiform layer [80]. Because the dSAC → PGC synapse is inhibitory, activation of the dSAC would inhibit the PGCs, which would counteract the PGC → SAC inhibition discussed above [83]. Furthermore, HDB cholinergic activity excites a minority of calretinin expressing cells that are excited by GABA release [84]. Together, these pathways likely provide inhibitory input to the glomeruli and MT output neurons. Our evidence indicates that HDB cholinergic neuron activation enhances both glomerular and MT dendritic field, which could not be explained by these pathways. Therefore, our evidence supports the model that the HDB → SAC circuit produces the disinhibitory effects we observed. This adds a layer of specificity to existing knowledge, which has mostly generalized the role of ACh without distinguishing between the contributions of different interneurons within the OB.

While our results demonstrate that cholinergic modulation via HDB projections can bias glomerular responses in a valence-dependent manner, it is important to recognize that perception and behavior also rely on integrative processing across multiple layers of the olfactory bulb and its cortical and subcortical targets. We found that AON and PIRc provide a higher proportion of input to SACs. These regions receive direct input from the OB and likely participate in reciprocal sensory loops to improve odor discrimination. While AON and PIRc have been implicated in goal directed behaviors, their precise role remains debated. One study reported that odor responses in the PIRc remained large unchanged during learning [48]. In contrast, more recent work demonstrated learning-related changes in PIRc representations [85]. Moreover, PIRc contributes to integrating cross-modal and learned associations, which can in turn modulate odor representations in the olfactory bulb [86,87]. Nevertheless, the HDB does not receive direct OB input, suggesting that its influence is more contextual and state-dependent rather than sensory-driven. While we cannot rule out potential contributions of these pathways to further modulate the SACs, our data indicate that the HDB → SAC → Glomeruli circuit substantially contributes to the modulation of odor processing during attention. Moreover, the HDB cholinergic system projects broadly across olfactory and non-olfactory brain regions, and its modulatory influence is likely distributed across these levels. Therefore, while our findings highlight a circuit mechanism by which attention and valence can shape early sensory representations in the olfactory bulb, further studies linking these glomerular dynamics to downstream processing and behavior are necessary to fully elucidate the multi-level transformation of sensory information into perception and action by the cholinergic system. Compared to direct excitation, a disinhibitory circuit may offer more flexibility in modulating and gating sensory responses. Inhibitory interactions between molecularly diverse interneurons produce distinct computational functions in the hippocampus, amygdala, and cerebral cortex under specific behavioral conditions [88–90]. Cholinergic impingement on dendritic disinhibition in primary sensory and motor cortices has been implicated in associative learning [91,92]. Our results indicate that cholinergic activity is associated with attention, raising the possibility that cholinergic mediated disinhibition may function in these cortical circuits to mediate attentional effect as well.

Our behavioral paradigm has biased associations between an odor signal and its reward value, thereby creating an imbalanced modulation inclined towards detection of rewarded odor. However, saliency can be dissociated from valence. A sensory signal associated with a potentially large negative outcome does not necessarily need to be suppressed. A signal predictive of the presence of a predator, for example, may need to be enhanced such that it can provide faster and more accurate information that assists the animal to escape. In this case, escaping capture may be presented as a potential reward such that salience of the predator signal should be enhanced rather than subdued. Although our current study does not test this scenario, the cholinergic signal is modified by the reward value. It is not difficult to imagine that the exhilaration from a successful escape could provide the reward signal needed to enhance the detection of a predator. Arguably, all top–down attention requires learning and reward contingency is likely built in the learning process. The disinhibitory circuit motif is prevalent throughout the brain and may provide a general mechanism for reward-based HDB cholinergic activity to generate selective attentional effect even though the cell types and neurotransmitters may vary.

## Materials and methods

### Ethics statement

All animal procedures were conducted in accordance with institutional and national guidelines for the care and use of laboratory animals, adhering to the NIH Guide for Care and Use of Animals. The experimental protocols were approved by the Institutional Animal Care and Use Committee at Stowers Institute for medical research under protocol number 2022-151.

### Animals

The *OMP-tTA* (Jackson laboratory, Stock no. 017754), *tetO-GCaMP2* (Stock no. 017755), *Chat-Cre* (Stock no. 006410), *DAT-Cre* (Stock no. 006660), *R-ChR2* (Stock no. 024109, Ai32), *C57BL/6* (Stock no. 000664) and *Cdhr1-Cre* (MMRRC, Stock no. 030952-UCD) mice were described previously. Male and female mice were at the age of 8–10 weeks at the start of training. All the animals were maintained in Lab Animal Services Facility at 12:12 h reversed light cycle and had access to food and water *ad libitum* until 3 days before behavioral training. All the behavior and functional imaging experiments were conducted during the dark light cycle.

### Odor delivery with olfactometer

Odor delivery was controlled by an automated olfactometer with custom written software developed in the National Instrument Labview [63]. The six odorants used are listed in S1 Table. All single compound chemicals were freshly prepared in mineral oil at 3:10^3^ (v/v). Odorants (100 mL/min) were then further diluted in carrier air with a maintained total flow rate (400 mL/min for all calcium imaging and behavior experiments) and were delivered next to the animal’s nose. A lick circuit detected contact which was used as an indicator to deliver water through a solenoid valve (Lee instruments).

### Animal behavior

Mice were placed on water-restriction (3 days) prior to behavioral training and maintained at >90% of their free drinking weight for the entire experiment. Mice were first acclimatized to head-fixation setup and learned to lick to collect water from the port before any odor exposure. During pre-training paradigm, mice were presented with CS+ odor with water reward. After mice reliably collected water (>80% of trials), they were given the full discrimination training paradigm which consisted of a pseudorandom delivery of CS+ or CS− odors with a total of 70–80 trials in a single session. Each trial was followed by a 15–40 s inter-trial interval. Response window was 3 s following a 0.3 s delay from odor delivery. Licks registered within the response window were awarded with 7.5 μl of water for a CS+ odor and punished with a brief air puff (400 mL/min, 2 s) for a CS− odor. If no response was registered, the trial was classified as a miss (CS+) or correct rejection (CS−). Pre attention probing, training was done with a 4 kHz auditory cue delivered for 0.5 s preceding the odors with a fixed foreperiod for all trials. A subset of animals (*N* = 14, Fig 1C) were tested with both delays in separate sessions to assess the impact of timing on attentional modulation. This design allowed us to examine how variations in the temporal relationship between the cue and the odor affect reaction times. The probe sessions were divided into 3 levels of odor duration (2, 1, 0.5 s) and auditory cue was removed for half of the total trials at each duration level. Only those sessions were used for further analysis which had <30% miss trials to ensure high motivation across session. To record sniffing, a thermocouple sensor was positioned close to the nose and minor adjustments made before each recording session until high signal-to-noise was achieved. Sampling was done at 1 kHz.

### Odor investigation assay

To assess odor investigation in freely moving mice, experiments were done as previously described [93]. Each animal was tested with CNO and saline delivery in two separate experiments with at least one week between tests. Air was passed through mineral oil and was delivered for 5 min in each trial followed by a 5 min interval. After four trials of air presentations, the testing odor was presented 4 times in the same interleaved sequence. The total time of nose poke during odor delivery was calculated from the IR beam break events at the odor delivery source.

### Stereotaxic surgeries

Mice were anesthetized by intraperitoneal injection of ketamine/xylazine cocktail (100 mg/kg, 10 mg/kg, respectively) and subcutaneous injection of buprenorphine (0.1 mg/kg) before being placed in a stereotactic frame. The body temperature was maintained at 37 °C with a heating pad. All viruses were injected at a final titer of 0.5–1 × 10^13^ vg/ml. For olfactory bulb experiments, a total of 4 small cavities (2 on each OB, anterior and posterior) were made using a dental drill for virus injection. At each injection site, a total volume of 250–350 nL was injected at multiple depths spanning 80−150 nm below the skull surface. After 3−4 weeks of virus injection, animals were anesthetized for cranial window preparation over the OB. For widefield glomeruli and MT fiber imaging, the skull above both OBs were thinned using a dental drill and a custom-made headbar was secured to the skull with dental cement. For optogenetic stimulation, a well was raised above the OBs using dental cement to secure optic fibers in place for stimulation. Following surgery, mice received buprenorphine for the next 2 days and recovered for at least 1 week before imaging or optogenetic experiments. For virus injection and subsequent GRIN lens implantation over HDB, a craniotomy was made using a dental drill for virus injection centered on the implantation coordinates (in mm): ML: 1.0; AP: 0.3. An amount of 350 nL of virus was injected at depths spanning DV: −5.45 to −5.25 on the left hemisphere. After 2 weeks from injection, a wider craniotomy (1–1.5 mm) was made at the previous injection site and the underlying cortex was aspirated. A tapered guiding needle (0.4 mm diameter) was then lowered at a speed of 20 μm/s to DV: −4.5. A 0.66 mm diameter and 7.6 mm length micro endoscope (1050-005442, Inscopix) was then inserted at a speed of 10 μm/s to DV: −5.0. The integrated lens was secured using dental cement together with a custom-made headbar. Following surgery, mice received buprenorphine for the next 4 days and recovered for at least 2 weeks before imaging experiments.

### Head-fixed imaging

For calcium imaging of glomeruli, generation of the GCaMP2 mice were described previously [31]. For imaging fiber responses and acetylcholine transients, AAV1-Syn-FLEX-jGCaMP7f (Addgene, 104492-AAV1), or AAV9-hSyn-DIO-Ach3 (WZ biosciences) respectively were injected bilaterally in OBs of *Cdhr1-cre* and *DAT-Cre* animals, respectively. Images were collected on an Olympus BX60WI microscope using 4× air lens (Olympus XLFLUOR4X/340, NA 0.28). The image was collected at 512 × 512 resolution with 2 × 2 bin with sampling rate at 10 Hz for SAC DREADDi and ACh GRAB experiments and 13.3 Hz unless otherwise stated. For recording HDB soma activity AAV1-syn-jGCaMP8m (Addgene, 162375-AAV1) was expressed unilaterally in *ChAT-cre* animals. Post GRIN lens implantation, a miniscope (Inscopix) was used to acquire images at 15 Hz sampling rate. For recording SAC soma activity, AAV1-Syn-FLEX-jGCaMP7f was expressed in *DAT-cre* animals. A 2-photon microscope was used. The image was collected by an Olympus 2-photon microscope (FLUOVIEW FVMPE-RS) with 940 nm excitation laser using 25× water lens (Olympus XLPLN25XWMP, NA 1.05). A resonate scanner with GaAsP detector was used for image collection at 512 × 512 resolution with 15 Hz sampling rate.

### Head-fixed optogenetics

For bilateral photoinhibition, Archaerhodopsin expressing short axon cells (AAV8-nEF-Con-Arch3.3-EYFP (Addgene, 137149-AAV8) in *DAT-Cre* mice) were bilaterally stimulated using optic fibers connected with a 560 nm LED source. The power at fiber tip was approximately 5–8 mW for all stimulation experiments. Control experiments were conducted on *DAT-Cre* animals that expressed a control GFP virus. For bilateral photoexcitation, Channelrhodopsin expressing cholinergic fibers (*ChAT-Cre; Rosa-ChR2*) in the olfactory bulb were bilaterally stimulated using optic fibers connected with a 488 nm LED source. The power at fiber tip was approximately 3–4 mW for all stimulation experiments. Control experiments were conducted on *ChAT-cre* animals that did not carry the *Rosa-ChR2* allele.

### DREADD inhibition and activation using CNO application

Inhibitory DREADDs were bilaterally expressed in short axon cells (AAV2-DIO-hM4D(Gi)-mCherry (Addgene, 44362-AAV2) in *DAT-Cre* mice) or *ChAT* fibers projecting to the OB (AAVrg-DIO-hM4D(Gi)-mCherry (Addgene, 44362-AAVrg) in *ChAT-Cre* mice) or HDB fibers projecting to the OB (AAVrg-EF1a-Cre (Addgene, 55636-AAVrg) together with AAV2-DIO-hM4D(Gi)-mCherry in *C57BL/6* mice). Excitatory DREADDs were bilaterally expressed in the HDB ChAT cells (AAV2-DIO-hM3D(Gq)-mCherry (Addgene, 44361-AAV2) in *ChAT-Cre* mice. Control experiments were conducted on *DAT-Cre* mice injected with AAV1-CAG-LSL-tdTomato (Addgene, 100048-AAV1) and *ChAT-Cre* mice injected with AAVrg-CAG-FLEX-tdTomato (Addgene, 51503-AAVrg) in the OB and AAV1-CAG-LSL-tdTomato in the HDB. Clozapine N-oxide (CNO, Abcam, ab141704) was diluted in 1× PBS and injected intraperitoneally at a dose of 2.5 mg/kg, 20 min before all behavior and imaging experiments.

### Histology

Mice were euthanized after anesthesia with urethane and perfused with 4% PFA in PBS. Brains were incubated in 4% PFA overnight at 4 °C, and coronal sections (60 μm) were cut by a vibratome (Leica VT1000S). Primary antibodies were diluted 1:1000 (Chicken anti-GFP (Abcam, ab13970), Rabbit anti-RFP (Rockland, 600-401-379), Goat anti-tdT (Origene, AB8181-200) or 1:400 (Rabbit anti-TH (MilliporeSigma, ab152), Goat anti-ChAT (ab144p)) with 1:20 Donkey serum and 1:20 DMSO in PBST (0.1% Triton X-100 in PBS) and applied on sections for incubation overnight at room temperature. Sections were washed with PBST 3 times for 30 min each before application of secondary antibodies diluted 1:1000 (Donkey anti-Chicken488 (Invitrogen, A78948), Donkey anti-Rabbit568 (A10042), Donkey anti-Rabbit 647 (A32795), Donkey anti-Goat568 (A11057), Donkey anti-Goat647 (A21447)) with 1:20 Donkey serum 1:20 DMSO and 1:1000 DAPI (1 μg/ml, MilliporeSigma, 268298) in PBST for incubation overnight at room temperature. Images were acquired using Olympus VS120 Virtual Slide Microscope at 10× magnification and binned 2 × 2 to confirm location as well as expression levels for jGCaMP7f, Ach3.0, GFP, mCherry, DREADDs, Archaerhodopsin, Channelrhodopsin and rabies tracing. Representative images were collected using Zeiss LSM-700 or Nikon AT-AT spinning disk confocal microscopes.

### Rabies tracing

*DAT-Cre* animals were injected with rabies helper AAV (AAV1- synP-FLEX-splitTVA-EGFP-B19G, Addgene, 52473-AAV1) to express glycoprotein (G) and the avian viral receptor TVA in SACs. At 3 weeks post AAV injection, EnvA pseudotyped and G-deleted rabies virus was injected at similar locations on the OB, and animal perfused 10 days post-injection [46]. Brain sections were imaged using Olympus Slidescanner and Nikon ATAT and analyzed in ImageJ.

### Quantification and statistical analysis

Significance was defined at *p* < 0.05 for all statistical tests used in this study. *, **, and *** indicate differences of *p* < 0.05, *p* < 0.01, and *p* < 0.001, respectively. OriginPro (OriginLab) and MATLAB (MathWorks) were used to calculate statistical significance. Summary of statistics are provided in S2 Table. One sample *t* test was used to determine if the mean values were significantly greater than zero. Wilcoxon rank-sum test (two-tailed) was used in two-group comparisons, and One-way repeated measures ANOVA followed by Tukey’s post hoc test was used in paired group comparisons. For multiple-group comparisons, two-way repeated measures ANOVA followed by Tukey’s post hoc test was performed to evaluate significance between pairs of groups. A mixed-design ANOVA was performed with Group as the between-subject factor and Condition as the within-subject factor. All data are presented as mean ± standard error of the mean.

### Analysis of behavioral data

Discrimination accuracy was calculated by averaging responses for CS+ trials and withholdings for CS− trials within the response window. Reaction time was calculated as the first lick detected in the response window. Training accuracy before probe session was calculated by a moving window average of 10 trials and the last window was chosen for classification of training level. For comparison between different trial types, we used rate-correct score (RCS) to determine task performance:


$$RCS_{\text{ij}}=\frac{Accuracy_{\text{ij}}}{RT_{\text{ij}}}$$

where *RT*_*ij*_ is the average reaction time for all correct trials performed by mouse *j* in condition *i*, and *Accuracy*_*ij*_ is the discrimination accuracy for mouse *j* in condition *i.* Improvement was calculated for each odor duration as:


$$Improvement\;(\%)=\frac{RS_{\text{cued}}-RCS_{\text{uncued}}}{RCS_{\text{uncued}}}\times 100$$

For optogenetics behavior analysis, stimulated trials (pre-odor and within odor period) were compared with non-stimulated within the same session using the above criteria.

### Analysis of sniffing data

Sniffing peaks and valleys were determined in MATLAB based on trace derivatives. Inhalation onset time was defined as the post-peak and pre-valley mid-point. Inter sniff intervals were estimated as the time between consecutive inhalation onsets. Instantaneous frequency was computed as the inverse of the inter-peak interval between successive inhalations, identified via peak. All sniff traces were manually checked for movement related signal loss mid-trial.

### Analysis of imaging data

Custom-written scripts in ImageJ and MATLAB (MathWorks) were used for image processing as described previously [31,93]. Briefly, ROIs were semi-manually defined using threshold response images. To identify the same ROI across multiple days, image stacks were *z*-projected to obtain mean intensity and aligned using angular rotation and translation in ImageJ. All spatially non-overlapping ROIs were pooled then the average response inside each ROI was extracted by batch processing in ImageJ. Using customized MATLAB software, a baseline was defined for each ROI using pre-stimulus time intervals and *dF*/*F* was calculated.

### Peak response

A 4 frame (300 ms) moving average was performed across the entire trace. Peak response of each ROI within the stimulus period was estimated as the maximum fluorescence detected on the average trace. This averaged trace was used to calculate mean and SEM across animals and plot the average responses. To calculate responsiveness of ROIs across behavior session, a threshold of 3 times the standard deviation was used for identified peaks. The area under the curve (AUC) was calculated using the trapz function in MATLAB to estimate the total response of each ROI during the stimulus period. The selectivity index for each ROI was calculated as the difference between peak responses between trial types divided by the SD of all responses.

### Temporal dynamics of response

Standard deviation for each ROI was defined using 2 s pre-stimulus interval and the first frame after which consecutive 4 frames exceeded 3*SD was used as the onset latency. Peak latency was estimated from the peak responses of the averaged trace for alignment of cell-odor traces.

### Significance of modulation for each ROI

To estimate significant modulation of activity or timing for each ROI (un-cued versus cued), all trials were pooled together, and trial identity shuffled 1,000 times to generate a null distribution of the modulation type. If the *p*-value from a *t* test followed by Benjamini–Hochberg FDR correction for multiple comparisons was less than 0.05, the ROI was counted as significantly modulated.

### Linear decoding

The activity trace of CS+ and CS− odors were extracted from both cued and un-cued trials, resulting in a binary trial map with labels indicating CS+ and CS− trials. A linear classification model (SVM) was used to classify trial types based on the activity of all ROIs. The model was trained using 5-fold cross-validation on the activity bins. The classification model was trained and evaluated in a sliding window manner across the entire duration of the trials. For each time bin, a subset of the data corresponding to that time window was used to train the SVM. The *k*-fold loss (*k*foldLoss) was used to quantify the classification error at each time bin. The decoding trace was obtained by plotting the classification accuracy over time, and the maximum accuracy during the odor presentation period was recorded for plotting.

### Logistic regression

All models were fit using MATLAB’s fitglm function with a binomial distribution and logit link. Each model was specified with odor activity level as the predictor and decision outcome (False alarm: 0 or Hit: 1) as the response variable. To evaluate the performance of each model relative to a baseline, null models were fitted ignoring any effects of activity for comparison against cued and un-cued conditions. Likelihood ratio test was employed to statistically compare each full model against its corresponding null model.

### Population modelling

We constructed a linear model using a set of stimuli (odor identity and auditory cue) and decision (lick/no-lick and reward) variables to capture modulation of signal across trained animals. To model the relationship between the predictors and neural activity, we employed lasso regression. This regularization technique is particularly useful when dealing with multicollinearity and introduces a penalty term and shrinks coefficients, effectively performing variable selection. We utilized 10-fold cross-validation to select the optimal value of the regularization parameter (lambda) that minimized the cross-validated mean squared error. This approach ensures that the model is neither overfitting nor underfitting the data.

### Calculation of partial *R*^2^

To assess the unique contribution of each predictor to the variance explained by the full model, we calculated the partial *R*^2^ [94]. For each neuron and time point, we fitted a full model using all predictors and reduced models excluding each predictor in turn. The partial *R*² for each predictor was then computed as the difference in *R*² between the full model and the reduced model, providing a measure of the unique contribution of each predictor to the variance in neural activity.

### Leaky-Integrate-and-fire network model

The network comprised of excitatory (E) and inhibitory (I) neurons that were stimulated with external inputs and interconnected via synapses that evolved over time. Our model incorporated a feature of clustered connectivity among both excitatory (E–E) and inhibitory (I–I) neuronal populations. This was intended to more accurately reflect the structured connectivity observed in sensory circuits [95]. The neuronal network was partitioned into 50 clusters, containing 10 excitatory and inhibitory neurons each. Within each cluster, neurons were more densely interconnected, representing higher synaptic coupling.

Each neuron’s membrane potential was simulated using the standard LIF model:


$$\tau \frac{dv}{dt}\;=\;-\left(v-v_{rest}\;\right)+I_{syn}+I_{ext}$$

where v is the membrane potential, τ is the membrane time constant, $v_{rest}$ is the resting membrane potential, $I_{syn}$ is the synaptic current, and $I_{ext}$ is the external current. When the membrane potential reached a threshold, the neuron fired, and *v* was reset to a reset potential. A refractory period was imposed post-firing during which the neuron could not fire again. Parameter values used are listed in S3 Table.

### Synaptic model

The synaptic currents were modeled using an exponential decay function to represent synaptic filtering. The synaptic strength was defined by a weight matrix *J*, where *J*(*i, j*) represented the strength of the connection from neuron *i* to neuron *j*. The synaptic dynamics were influenced by the firing of presynaptic neurons, with the synaptic conductance updated at each time step.

We incorporated a synaptic plasticity mechanism based on Hebbian learning principles.


$$\text{Long}-\text{Term Potentiation }(\text{LTP}):\Delta W_{ij\;}=\;{syn}_{+}e^{\left(-\frac{\left|t_{i}-t_{j}\right|}{\tau_{r}}\right)}\text{ for }t_{i}<t_{j}$$

$$\text{Long}-\text{Term Depression }(\text{LTD}):\Delta W_{ij\;}=\;-{syn}_{-}e^{\left(-\frac{\left|t_{i}-t_{j}\right|}{\tau_{r}}\right)}\text{ for }t_{j}<t_{i}$$

The synaptic weights, represented by the *J*(E–I) and *J*(I–E) matrices for excitatory-to-inhibitory and inhibitory-to-excitatory connections, respectively, were adjusted based on the correlation of neuronal firing patterns observed over the course of each iteration. If an inhibitory neuron fired within a predefined time window after an excitatory neuron, the synaptic weight between them was increased. This increase was modulated by the timing difference between firings, with a decay factor ($\tau_{r}$) and a learning rate (${syn}_{+}$). Conversely, if the inhibitory neuron fired prior to the excitatory neuron, the synaptic weight was decreased by rate ${syn}_{-}$.

### External stimulation

The network was stimulated externally with time-varying stimuli profiles. Each stimulus was modeled as a percentage change in the baseline current for a selective group of neurons. A 10% of total excitatory clusters were selective for either one of the conditioned stimuli. Top–down disinhibition during delivery of CS+, CS− or attention cue acted as an inhibitory bias upon 80% of all inhibitory neurons. During training, CS+ induced disinhibitory was simulated with a stronger inhibitory bias than the CS− stimulus, which created preferential increase in synaptic weights based on differential disinhibition for the two stimuli, without changing the selectivity of the disinhibition. Attention cue was simulated as an inhibitory bias prior to delivery of the conditioned stimuli.

## Supporting information

S1 Fig Behavior training and dynamics of MTC dendritic activity in cued-attention paradigm. **(A)** Lick events recorded for CS+ and CS− odors at different training sessions. Blue and brown shading show delivery of CS+ and CS− odors, respectively. **(B)** Training accuracy across time (blocks of 10 CS± trials) and across multiple sessions. **(C)** Lick events recorded in a session for cued trials across time. Right: Lick events pre-odor delivery across multiple training sessions prior to attention test session. ***P < 0.001, one-way repeated measures ANOVA followed by Tukey’s post hoc test (*q*(33) = 8.66, *p* < 0.0001). **(D)** AUC for pre-odor period (*t* = −1 to 0 s) for all task conditions (*n* = 19 animals). **P < 0.01, two-way repeated measures ANOVA followed by Tukey’s post hoc test (*q*(18) = 4.43, *p* = 0.0057). **(E)** Bar plots show the percentage of glomeruli exhibiting a change in response for cued versus un-cued trials based on glomerular response during foreperiod (*t* = −1 to 0 s) across odors. **(F, G)** Distribution of difference between peak responses **(F)** and onset latency **(G)** of cued versus un-cued trials for 0.5 s odor delivery. **(H)** Difference of activity between cued and un-cued trials (*ΔΔF*/*F*) for each glomerulus (*n* = 1,066 glomeruli from 19 animals) for 2 s odor delivery (compared to 0.5 s in Fig 1). **(I)** Average glomerular activity trace for un-cued (left) and cued odor stimulation (right). Orange shading shows cue presentation. Gray shading shows odor delivery. Data are mean ± SEM. **(J)** Area under the curve (AUC) for odor period (*t* = 0 to 2 s). **P < 0.01, two-way repeated measures ANOVA followed by Tukey’s post hoc test (*q*(18) = 4.48, *p* = 0.0053). **(K, L)** Distribution of difference between peak responses **(K)** and onset latency **(L)** of cued versus un-cued trials for 0.5 s odor delivery. **(M)** Heatmaps show the difference in dendritic responses of MTCs activity between cued and un-cued trials (*ΔΔF*/*F*) for CS+ (left), CS− (right) odors in jGCaMP7f injected *Cdhr1-Cre* animals (*n* = 19 animals). **(N, O)** Area under the curve (AUC) for pre-odor (*t* = −1 to 0 s; *q*(18) = 9.98, *p* < 0.0001) and odor period (*t* = 0 to 0.5 s; *q*(18) = 5.92, *p* = 0.00055). ***P < 0.001, two-way repeated measures ANOVA followed by Tukey’s post hoc test. **(P, Q)** Distribution of difference of activity **(P)** and onset latency **(Q)** between cued and un-cued trials for the ROIs. Data for this figure are provided in S1 Data.
(TIF)

S2 Fig Anatomical and functional validation of SAC and HDB circuit manipulations. **(A, B)** Spatial spread of virus infection over the dorsal OB. **(C)** Density observed across 9 DREADDi injected animals used for behavior and glomeruli imaging studies reported in Fig 2. **(D)** Accuracy and RT differences between un-cued trials of CNO and saline injected (Gi; purple, *n* = 9) and mCherry (mCh; gray, *n* = 8) animals. Mixed-design ANOVA followed by Tukey’s post hoc test. (Gi-saline versus CNO, accuracy: *q*(15) = 0.65, *p* = 0.96; RT: *q*(15) = 0.916, *p* = 0.91). **(E)** Decoding accuracy based on glomerular responses across cued versus un-cued trials in Gi mice for Saline (left) and CNO (right) injected sessions. **(F)** Representative images of rabies infected cells (green) that project to SACs. SACs receive signals from anterior olfactory nucleus (AON), piriform cortex (PIR), zona incerta (ZI), locus coeruleus (LC) but not dorsal raphe nucleus (DR). No colocalization with cholinergic antibody (red) was found in the SAC projecting neurons of these regions (*n* = 3 animals), scale bar: 100 µm. **(G)** Total number of cells that project to SACs from brain areas (left). Number of SAC projecting cells that are ChAT positive (right). **(H)** Strategy for chemogenetic inhibition of basal forebrain projections. BL/6 animals were injected with DREADDs or mCh virus in the HDB. **(I)** Accuracy (mCh: *q*(12) = 3.62, *p* = 0.017; Gi: *q*(12) = 1.58, *p* = 0.28) and RT (mCh: *q*(12) = 3.81, *p* = 0.019; Gi: *q*(12) = 0.47, *p* = 0.74) in cued versus un-cued trials post CNO injections. *P < 0.05, Mixed-design ANOVA followed by Tukey’s post hoc test. **(J)** RCS improvement of mCh and Gi mice after CNO injection. *P < 0.05, **P < 0.01, one sample *t* test and two sample *t* test (two-tailed; *t*(12) = 3.15, *p* = 0.0083). **(K, L)** Example of spatial spread of retrograde virus infection in HDB **(K)** and quantification across 10 animals **(L)** used for behavioral and imaging study in Fig 2. **(M)** Accuracy and RT differences between un-cued trials of CNO and saline injected (Gi; purple, *n* = 10) and mCherry (mCh; gray, *n* = 9) animals. Mixed-design ANOVA followed by Tukey’s post hoc test. (Gi-saline versus CNO, accuracy: *q*(17) = 0.19, *p* = 0.99; RT: *q*(17) = 2.39, *p* = 0.35). **(N)** Strategy for optogenetic inhibition of cholinergic projections. ChAT-Cre animals were injected with Arch or GFP (left). LED stimulation from *t* = −1 to 0 s, cue delivered from *t* = −0.5 to 0 s, odor delivered from *t* = 0 to 0.5 s. RCS improvement of GFP (*n* = 10) and Arch (*n* = 10) pre-proficient mice after cue and pre-odor stimulation (right). *P < 0.05, one sample *t* test and two sample *t* test (two-tailed; *t*(18) = 2.33, *p* = 0.031). Data for this figure are provided in S1 Data.
(TIF)

S3 Fig Attention biases CS+ odors through a HDB to SAC circuit. **(A, B)** Example implantation site over HDB. Area under the curve (AUC) for pre-odor **(A)** and odor period **(B)**. *P < 0.05, two-way repeated measures ANOVA followed by Tukey’s post hoc test (*q*(7) = 3.2, *p* = 0.012). **(C, D)** Same as **(A, B)**, but for activities recorded from SACs in DAT-Cre mice (*n* = 6 animals). Data for this figure are provided in S1 Data.
(TIF)

S4 Fig Attention engages sniffing pre- but not post-odor presentation. **(A)** Cumulative distribution of inter sniff intervals in cued trials. Cued (orange) and un-cued (black) trials were plotted for untrained (*q*(15) = 3.57, *p* = 0.023), < 70% (“Amateur” *q*(8) = 2.45, *p* = 0.12), 70%–80% (“Pre-proficient” *q*(18) = 6.18, *p* = 3.68 × 10^−4^) and >80% training accuracy (“Proficient” *q*(32) = 0.39, *p* = 0.78). *P < 0.05, ***P < 0.001, one-way repeated measures ANOVA followed by Tukey’s post hoc test. Data are mean ± SEM. **(B)** Left panel: Power spectral density (PSD) plot obtained for pre-odor period (*t* = −1 to 0 s). Cued (orange) and un-cued (black) trials were plotted for pre-proficient animals. Right panel: Bar plots show mean power at 3–6 Hz in the sniff recording. *P < 0.05, one-way repeated measures ANOVA followed by Tukey’s post hoc test (*q*(18) = 3.67, *p* = 0.018). **(C)** Average number of sniffs detected across time bins prior to odor delivery. **(D, E)** Same as **(B, C)** but for proficient animals (*q*(32) = 1.7, *p* = 0.21). **(F)** Bar plots show delay of first inhalation after odor presentation in cued versus un-cued trials. No difference was observed between odors of opposing valence (*q*(18) = 0.22, *p* = 0.87). **(G)** Bar plots show inter sniff delay within the 1st second of odor delivery in pre-proficient animals (*q*(18) = 0.08, *p* = 0.95). **(H)** Cumulative distribution of sniffs with CS+ and CS− odor presentation. **(I–K)** Same as **(F–H)**, but for proficient animals (First inhalation: *q*(32) = 0.62, *p* = 0.66; Inter sniff delay: *q*(32) = 0.53, *p* = 0.7). **(L)** Correct odor discrimination decisions in cued (solid line) versus un-cued (dotted line) trials across pre-test performance accuracy and odor delivery duration (<70% *n* = 9, hit rate 2 s: *P < 0.05, 1 s: N.S. and 0.5 s: N.S.; correct rejection rate 2 s: *P < 0.05, 1 s: N.S. and 0.5 s: N.S.; 70%–80%, *n* = 19, hit rate 2 s: N.S., 1 s: N.S. and 0.5 s: N.S.; correct rejection rate 2 s: N.S., 1 s: *P < 0.05 and 0.5 s: ***P < 0.001; 80%–90%, *n* = 14, hit rate 2 s: N.S., 1 s: N.S. and 0.5 s: N.S.; correct rejection rate 2 s: N.S., 1 s: *P < 0.05 and 0.5 s: N.S.; >90%, *n* = 17, hit rate 2 s: N.S., 1 s: N.S. and 0.5 s: N.S.; correct rejection rate 2 s: *P* = 0.07, 1 s: N.S. and 0.5 s: N.S. Blue: hit responses, Brown: correct rejections. **(M)** Average reaction times for CS+ odor in cued (solid line) versus un-cued (dotted line) trials across pre-test performance accuracy (<70%, 2 s: N.S., 1 s: N.S. and 0.5 s: N.S.; 70%–80%, 2 s: N.S., 1 s: N.S. and 0.5 s: **P < 0.01; 80%–90%, 2 s: N.S., 1 s: N.S. and 0.5 s: *P < 0.05; >90%, 2 s: N.S., 1 s: N.S. and 0.5 s: N.S.). **(N)** Performance distribution prior to binning. Olive-color shading highlights data from the pre-proficient group. Data for this figure are provided in S1 Data.
(TIF)

S5 Fig Dynamics of glomerular and ACh activity in trained animals. **(A, B)** Heatmaps of response trace **(A)** and distribution of difference of activity **(B)** between cued and un-cued trials (*ΔΔF*/*F*) for each glomerulus in *n* = 11 proficient animals. **(C)** Distribution of difference in onset latency between cued and un-cued trials for each glomerulus in proficient animals. **(D)** AUC for pre-odor period (*t* = −1 to 0 s) for all task conditions of proficient animals. N.S., two-way repeated measures ANOVA followed by Tukey’s post hoc test (*q*(10) = 0.12, *p* = 0.3). **(E)** Strategy for imaging ACh transients upon SACs. **(F, G)** Heatmaps show differences in ACh activity between CS+ and CS− odors (*ΔΔF*/*F*) for the same ROI in un-cued (left) and cued (right) trials for pre-proficient **(F)** and proficient **(G)** animals (*n* = 13). **(H)** Heatmaps show ACh activity segregated according to decision; odor was delivered from time = 0 to 0.5 s. **(I)** Violin plots show ACh activity as AUC segregated by decisions. ***P < 0.001, two-way repeated measures ANOVA followed by Tukey’s post hoc test (un-cued; *q*(382) = 7.7, *p* < 0.0001; cued: *q*(382) = 15.66, *p* < 0.0001). **(J)** Logistic regression curves show the probability of a Hit response as a function of odor-evoked ACh activity for cued (orange) and un-cued (gray) trials. Dotted line represents null distribution. **(K)** Instantaneous sniffing frequency from a proficient mouse before (Saline) and after (CNO) HDB activation. ***P < 0.001, two-way repeated measures ANOVA followed by Tukey’s post hoc test (*q*(9) = 8.22, *p* = 5.4 × 10^−4^). Data for this figure are provided in S1 Data.
(TIF)

S6 Fig Activation of HDB enhances neutral odor investigation in naïve animals. **(A)** Setup for odor investigation assay. An odor is delivered in intervals of 5 min and investigation events are registered from nose-poke IR beam break. Right: Strategy for testing role of HDB in odor investigation. **(B)** Normalized investigation for 4 intervals of air delivery with subsequent intervals of odor delivery from mice without (Saline) and with (CNO) HDB activation (*n* = 9 animals, left; *q*(7) = 3.93, *p* = 0.027) and mice injected with control virus (*n* = 8 animals, right; *q*(7) = 1.78, *p* = 0.24). *P < 0.05, one-way repeated measures ANOVA followed by Tukey’s post hoc test. **(C)** Comparison of odor investigation across different viral conditions using investigation index. *P < 0.05, **P < 0.01, one sample *t* test and two-way ANOVA followed by Tukey’s post hoc test (Gq-Saline versus CNO: *q*(39) = 4.8, *p* = 0.008). **(D)** Same as **(B)** but quantification of the delay period between the clean air or odor presentation (Gq: *q*(7) = 3.37, *p* = 0.048; mCh: *q*(7) = 0.36, *p* = 0.8). Arrows represent post odor delivery intervals. *P < 0.05, one-way repeated measures ANOVA followed by Tukey’s post hoc test. Data for this figure are provided in S1 Data.
(TIF)

S7 Fig Selective sensory remodeling underlies attentional adaptation. **(A)** Average activity of excitatory (left) and inhibitory clusters (right) to odor stimulation without implementing learning rules. **(B)** Decoding accuracy of stimulus identity with or without the attention cue in the absence of valence-based learning. Top–down attention was modeled from *t* = −0.5 to 0 s, input signal and top–down odor bias was modeled from *t* = 0 to 0.5 s. Right: Accuracy in decoding with or without attention cue for 16 pairs of input patterns. One-way repeated measures ANOVA followed by Tukey’s post hoc test (*q*(15) = 2.16, *p* = 0.14). **(C)** Changes in synaptic weights across excitatory and inhibitory neurons after training. Blue and brown shading show CS+ and CS− inputs respectively, gray shading shows inhibitory neurons common to both inputs. Data for this figure are provided in S1 Data.
(TIF)

S1 Table Odorants used in this study. (XLSX)

S2 Table Summary of statistics. (XLSX)

S3 Table Parameters for leaky-integrate-and-fire neural network. (XLSX)

S1 Data All tabulated data. (XLSX)

## Abbreviations

ITI: inter-trial interval
OSN: olfactory sensory neuron
MTC: mitral/tufted cell
OB: olfactory bulb
RCS: rate correct score
RT: reaction time
SAC: short axons cell
HDB: horizontal limb of the diagonal band.
