# Supplementary figures and images for: Basal forebrain cholinergic input mediates adaptive attention allocation to enhance olfactory discrimination
Source: PLoS Biol. 2025 Sep 16;23(9):e3003374. doi: 10.1371/journal.pbio.3003374 (PMC12440206; doi:10.1371/journal.pbio.3003374)

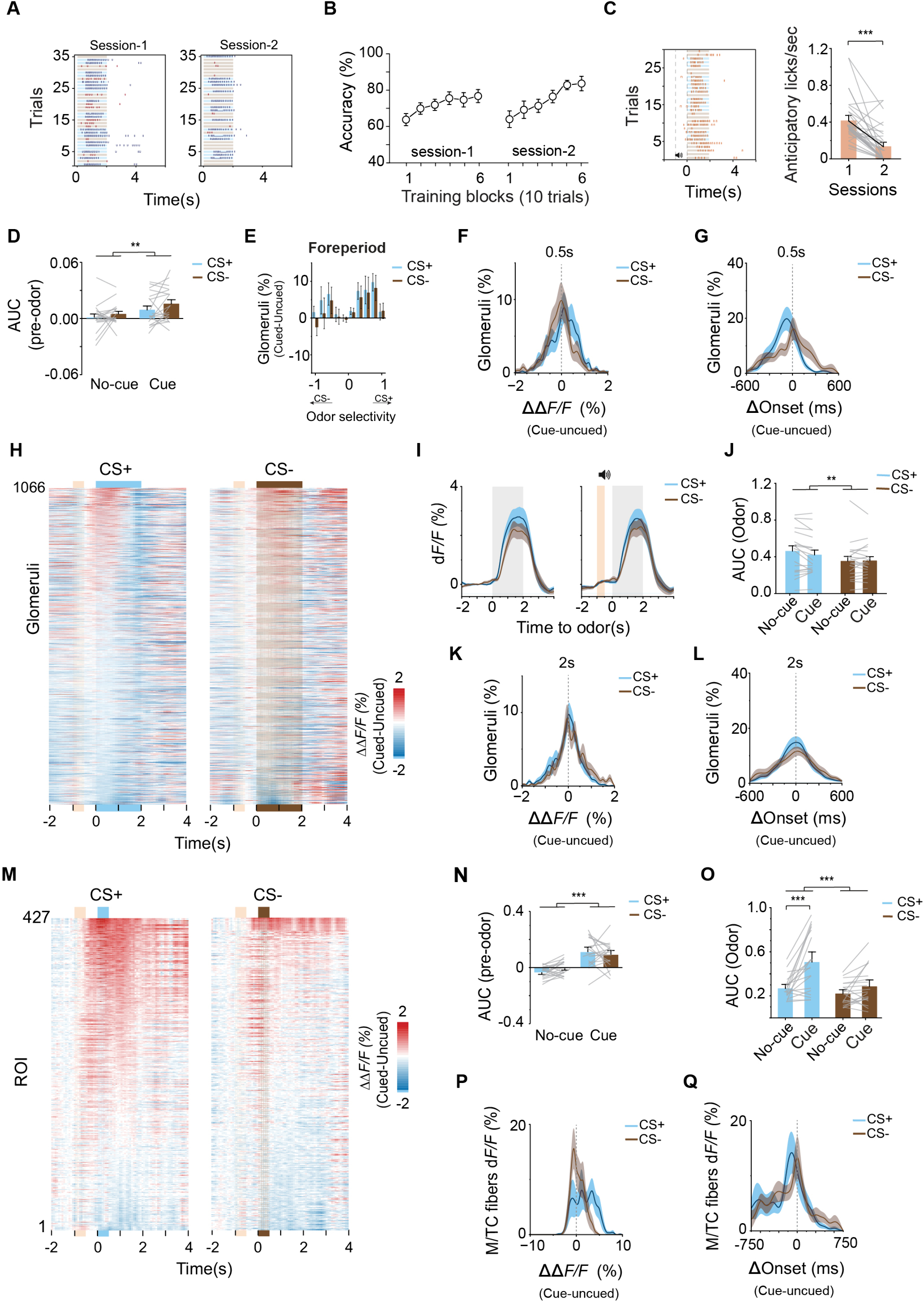

Supplement: S1 Fig — (A) Lick events recorded for CS+ and CS− odors at different training sessions. Blue and brown shading show delivery of CS+ and CS− odors, respectively. (B) Training accuracy across time (blocks of 10 CS± trials) and across multiple sessions. (C) Lick events recorded in a session for cued trials across time. Right: Lick events pre-odor delivery across multiple training sessions prior to attention test session. ***P < 0.001, one-way repeated measures ANOVA followed by Tukey’s post hoc test (q(33) = 8.66, p < 0.0001). (D) AUC for pre-odor period (t = −1 to 0 s) for all task conditions (n = 19 animals). **P < 0.01, two-way repeated measures ANOVA followed by Tukey’s post hoc test (q(18) = 4.43, p = 0.0057). (E) Bar plots show the percentage of glomeruli exhibiting a change in response for cued versus un-cued trials based on glomerular response during foreperiod (t = −1 to 0 s) across odors. (F, G) Distribution of difference between peak responses (F) and onset latency (G) of cued versus un-cued trials for 0.5 s odor delivery. (H) Difference of activity between cued and un-cued trials (ΔΔF/F) for each glomerulus (n = 1,066 glomeruli from 19 animals) for 2 s odor delivery (compared to 0.5 s in Fig 1). (I) Average glomerular activity trace for un-cued (left) and cued odor stimulation (right). Orange shading shows cue presentation. Gray shading shows odor delivery. Data are mean ± SEM. (J) Area under the curve (AUC) for odor period (t = 0 to 2 s). **P < 0.01, two-way repeated measures ANOVA followed by Tukey’s post hoc test (q(18) = 4.48, p = 0.0053). (K, L) Distribution of difference between peak responses (K) and onset latency (L) of cued versus un-cued trials for 0.5 s odor delivery. (M) Heatmaps show the difference in dendritic responses of MTCs activity between cued and un-cued trials (ΔΔF/F) for CS+ (left), CS− (right) odors in jGCaMP7f injected Cdhr1-Cre animals (n = 19 animals). (N, O) Area under the curve (AUC) for pre-odor (t = −1 to 0 s; q(18) = 9.98, p < 0.0001) [file pbio.3003374.s001.tif]

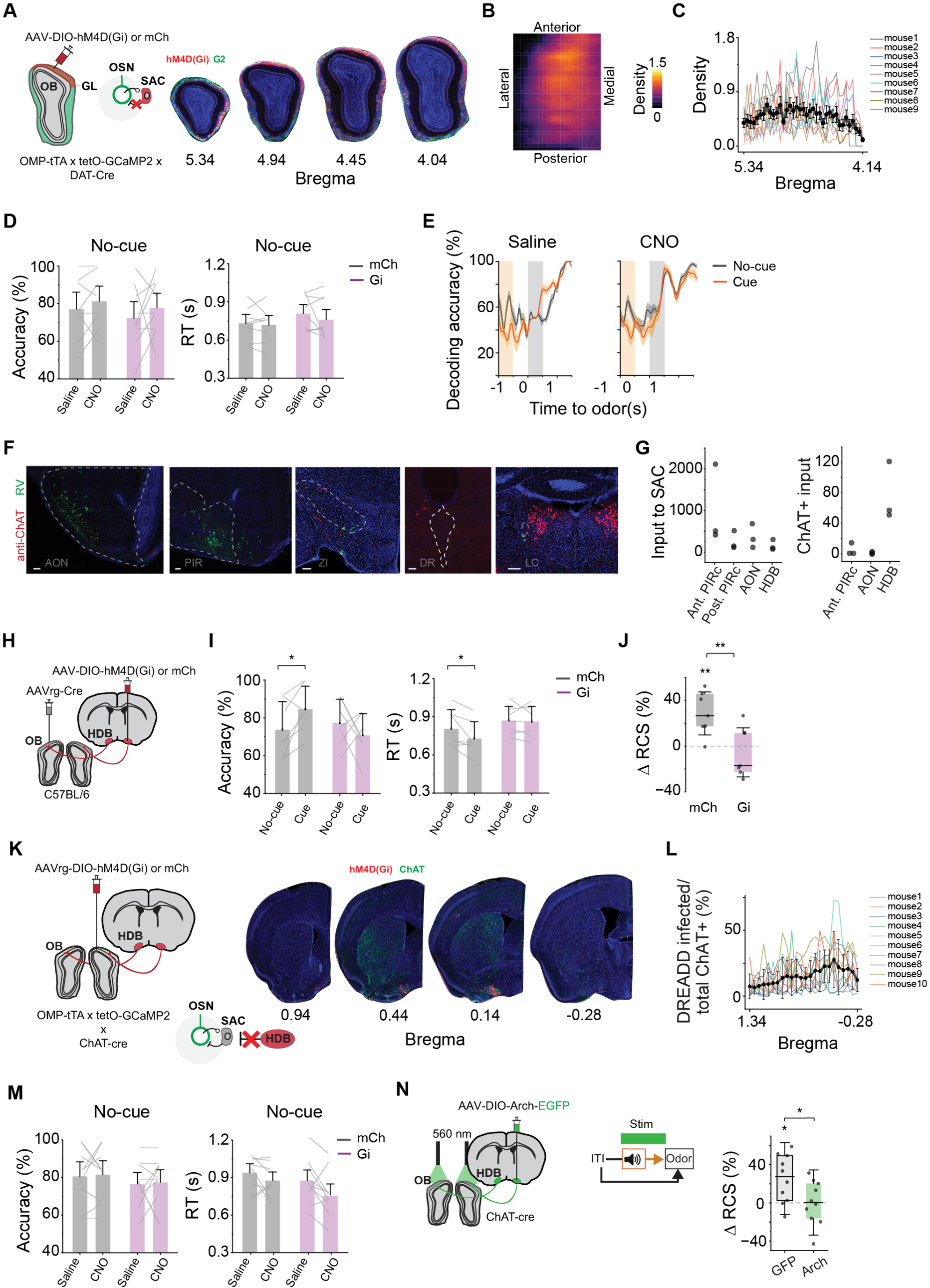

Supplement: S2 Fig — (A, B) Spatial spread of virus infection over the dorsal OB. (C) Density observed across 9 DREADDi injected animals used for behavior and glomeruli imaging studies reported in Fig 2. (D) Accuracy and RT differences between un-cued trials of CNO and saline injected (Gi; purple, n = 9) and mCherry (mCh; gray, n = 8) animals. Mixed-design ANOVA followed by Tukey’s post hoc test. (Gi-saline versus CNO, accuracy: q(15) = 0.65, p = 0.96; RT: q(15) = 0.916, p = 0.91). (E) Decoding accuracy based on glomerular responses across cued versus un-cued trials in Gi mice for Saline (left) and CNO (right) injected sessions. (F) Representative images of rabies infected cells (green) that project to SACs. SACs receive signals from anterior olfactory nucleus (AON), piriform cortex (PIR), zona incerta (ZI), locus coeruleus (LC) but not dorsal raphe nucleus (DR). No colocalization with cholinergic antibody (red) was found in the SAC projecting neurons of these regions (n = 3 animals), scale bar: 100 µm. (G) Total number of cells that project to SACs from brain areas (left). Number of SAC projecting cells that are ChAT positive (right). (H) Strategy for chemogenetic inhibition of basal forebrain projections. BL/6 animals were injected with DREADDs or mCh virus in the HDB. (I) Accuracy (mCh: q(12) = 3.62, p = 0.017; Gi: q(12) = 1.58, p = 0.28) and RT (mCh: q(12) = 3.81, p = 0.019; Gi: q(12) = 0.47, p = 0.74) in cued versus un-cued trials post CNO injections. *P < 0.05, Mixed-design ANOVA followed by Tukey’s post hoc test. (J) RCS improvement of mCh and Gi mice after CNO injection. *P < 0.05, **P < 0.01, one sample t test and two sample t test (two-tailed; t(12) = 3.15, p = 0.0083). (K, L) Example of spatial spread of retrograde virus infection in HDB (K) and quantification across 10 animals (L) used for behavioral and imaging study in Fig 2. (M) Accuracy and RT differences between un-cued trials of CNO and saline injected (Gi; purple, n = 10) and mCherry (mCh; gray, n = 9) animals. Mixed- [file pbio.3003374.s002.tif]

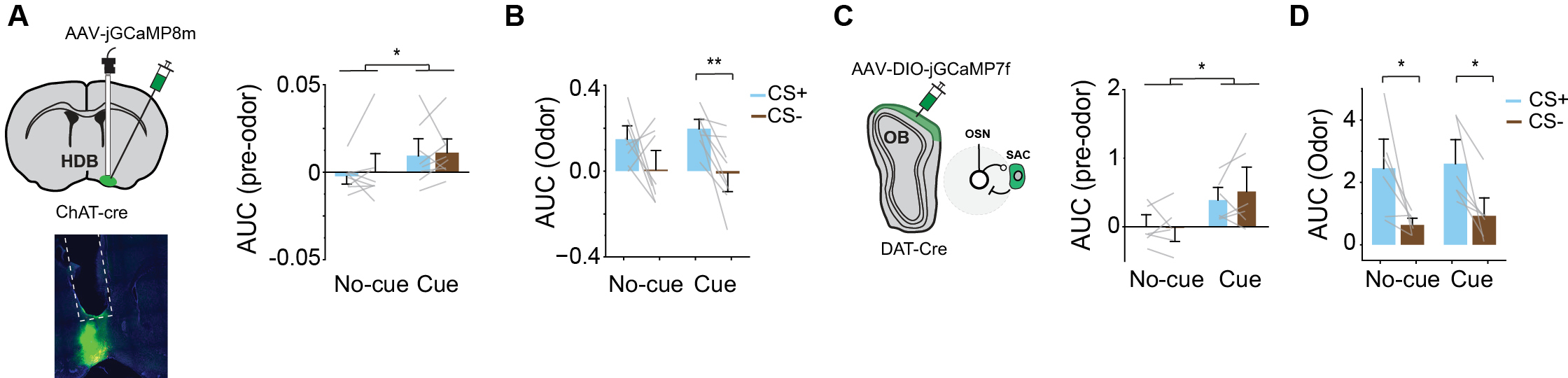

Supplement: S3 Fig — (A, B) Example implantation site over HDB. Area under the curve (AUC) for pre-odor (A) and odor period (B). *P < 0.05, two-way repeated measures ANOVA followed by Tukey’s post hoc test (q(7) = 3.2, p = 0.012). (C, D) Same as (A, B), but for activities recorded from SACs in DAT-Cre mice (n = 6 animals). Data for this figure are provided in S1 Data. (TIF) [file pbio.3003374.s003.tif]

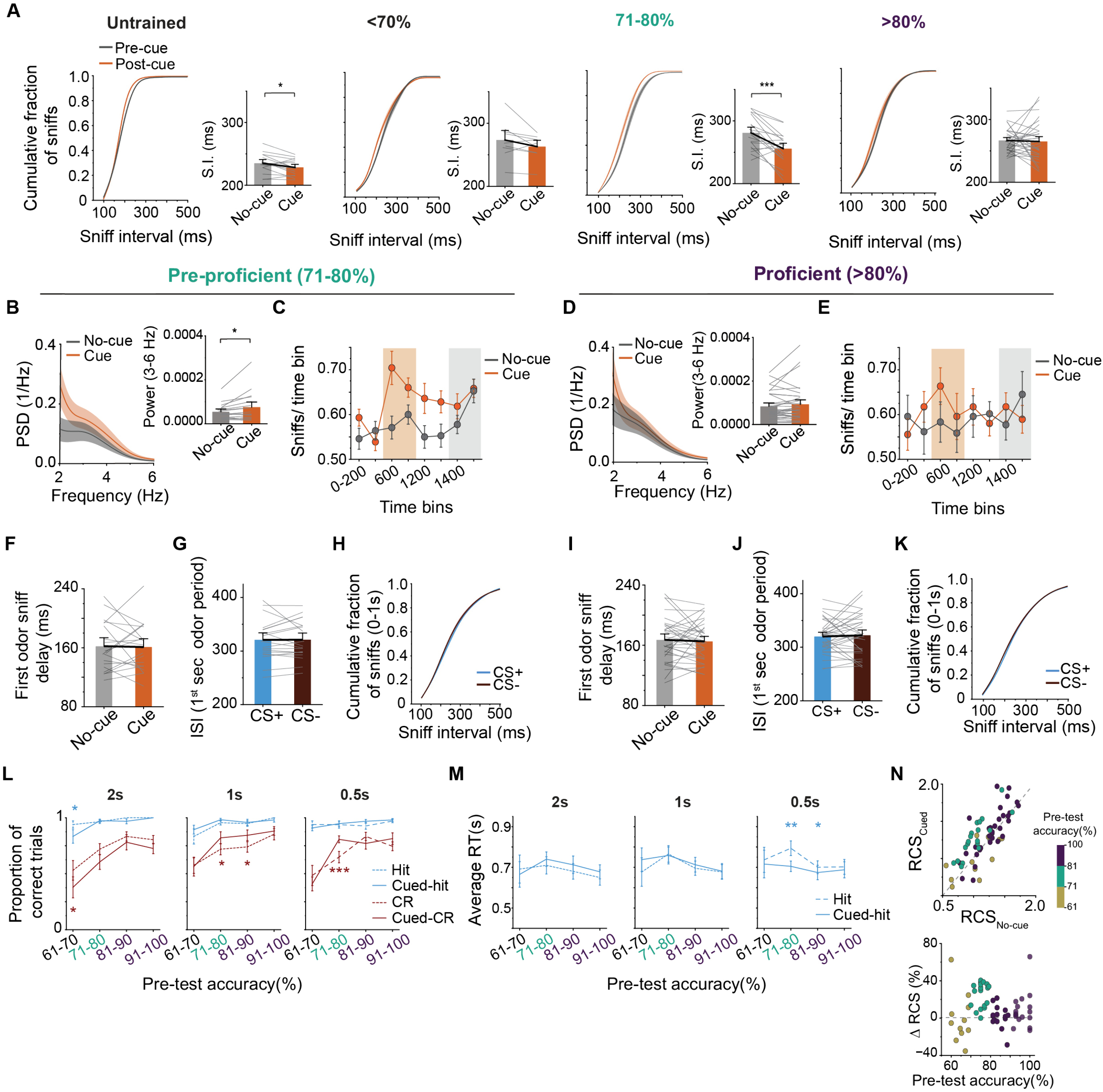

Supplement: S4 Fig — (A) Cumulative distribution of inter sniff intervals in cued trials. Cued (orange) and un-cued (black) trials were plotted for untrained (q(15) = 3.57, p = 0.023), < 70% (“Amateur” q(8) = 2.45, p = 0.12), 70%–80% (“Pre-proficient” q(18) = 6.18, p = 3.68 × 10^−4^) and >80% training accuracy (“Proficient” q(32) = 0.39, p = 0.78). *P < 0.05, ***P < 0.001, one-way repeated measures ANOVA followed by Tukey’s post hoc test. Data are mean ± SEM. (B) Left panel: Power spectral density (PSD) plot obtained for pre-odor period (t = −1 to 0 s). Cued (orange) and un-cued (black) trials were plotted for pre-proficient animals. Right panel: Bar plots show mean power at 3–6 Hz in the sniff recording. *P < 0.05, one-way repeated measures ANOVA followed by Tukey’s post hoc test (q(18) = 3.67, p = 0.018). (C) Average number of sniffs detected across time bins prior to odor delivery. (D, E) Same as (B, C) but for proficient animals (q(32) = 1.7, p = 0.21). (F) Bar plots show delay of first inhalation after odor presentation in cued versus un-cued trials. No difference was observed between odors of opposing valence (q(18) = 0.22, p = 0.87). (G) Bar plots show inter sniff delay within the 1st second of odor delivery in pre-proficient animals (q(18) = 0.08, p = 0.95). (H) Cumulative distribution of sniffs with CS+ and CS− odor presentation. (I–K) Same as (F–H), but for proficient animals (First inhalation: q(32) = 0.62, p = 0.66; Inter sniff delay: q(32) = 0.53, p = 0.7). (L) Correct odor discrimination decisions in cued (solid line) versus un-cued (dotted line) trials across pre-test performance accuracy and odor delivery duration (<70% n = 9, hit rate 2 s: *P < 0.05, 1 s: N.S. and 0.5 s: N.S.; correct rejection rate 2 s: *P < 0.05, 1 s: N.S. and 0.5 s: N.S.; 70%–80%, n = 19, hit rate 2 s: N.S., 1 s: N.S. and 0.5 s: N.S.; correct rejection rate 2 s: N.S., 1 s: *P < 0.05 and 0.5 s: ***P < 0.001; 80%–90%, n = 14, hit rate 2 s: N.S., 1 s: N.S. and 0.5 s: N.S.; correct rejection rate 2 s: N.S. [file pbio.3003374.s004.tif]

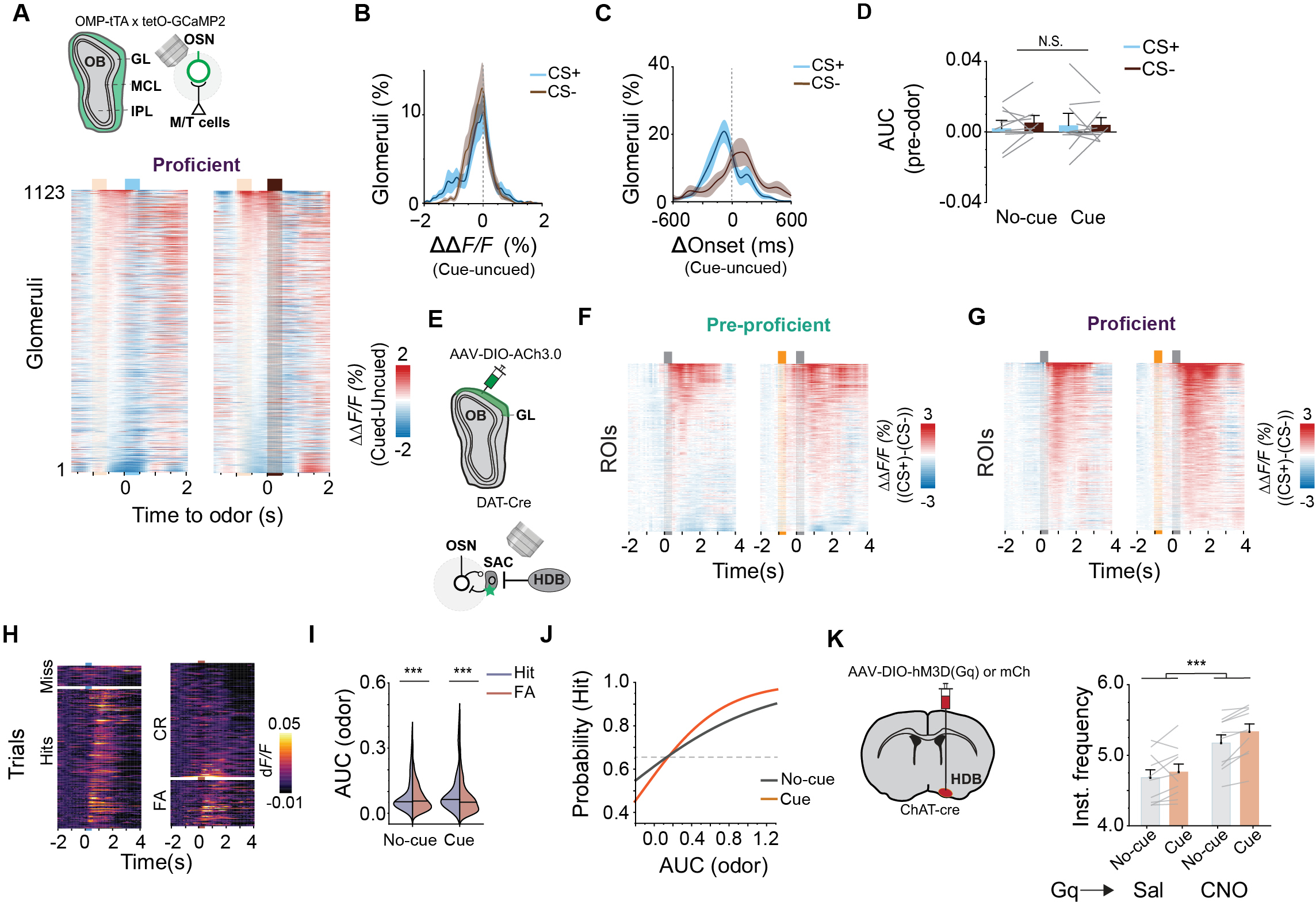

Supplement: S5 Fig — (A, B) Heatmaps of response trace (A) and distribution of difference of activity (B) between cued and un-cued trials (ΔΔF/F) for each glomerulus in n = 11 proficient animals. (C) Distribution of difference in onset latency between cued and un-cued trials for each glomerulus in proficient animals. (D) AUC for pre-odor period (t = −1 to 0 s) for all task conditions of proficient animals. N.S., two-way repeated measures ANOVA followed by Tukey’s post hoc test (q(10) = 0.12, p = 0.3). (E) Strategy for imaging ACh transients upon SACs. (F, G) Heatmaps show differences in ACh activity between CS+ and CS− odors (ΔΔF/F) for the same ROI in un-cued (left) and cued (right) trials for pre-proficient (F) and proficient (G) animals (n = 13). (H) Heatmaps show ACh activity segregated according to decision; odor was delivered from time = 0 to 0.5 s. (I) Violin plots show ACh activity as AUC segregated by decisions. ***P < 0.001, two-way repeated measures ANOVA followed by Tukey’s post hoc test (un-cued; q(382) = 7.7, p < 0.0001; cued: q(382) = 15.66, p < 0.0001). (J) Logistic regression curves show the probability of a Hit response as a function of odor-evoked ACh activity for cued (orange) and un-cued (gray) trials. Dotted line represents null distribution. (K) Instantaneous sniffing frequency from a proficient mouse before (Saline) and after (CNO) HDB activation. ***P < 0.001, two-way repeated measures ANOVA followed by Tukey’s post hoc test (q(9) = 8.22, p = 5.4 × 10^−4^). Data for this figure are provided in S1 Data. (TIF) [file pbio.3003374.s005.tif]

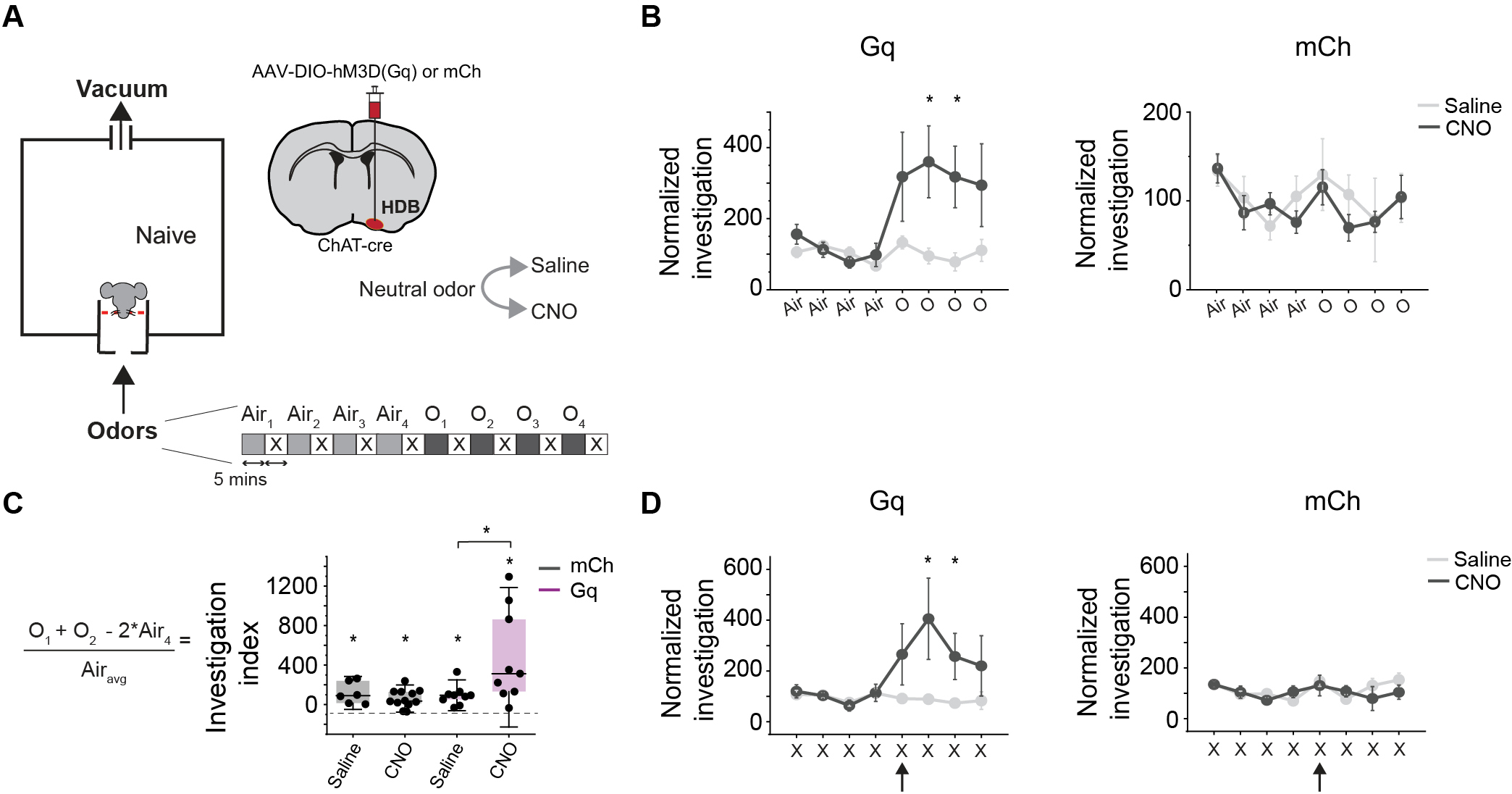

Supplement: S6 Fig — (A) Setup for odor investigation assay. An odor is delivered in intervals of 5 min and investigation events are registered from nose-poke IR beam break. Right: Strategy for testing role of HDB in odor investigation. (B) Normalized investigation for 4 intervals of air delivery with subsequent intervals of odor delivery from mice without (Saline) and with (CNO) HDB activation (n = 9 animals, left; q(7) = 3.93, p = 0.027) and mice injected with control virus (n = 8 animals, right; q(7) = 1.78, p = 0.24). *P < 0.05, one-way repeated measures ANOVA followed by Tukey’s post hoc test. (C) Comparison of odor investigation across different viral conditions using investigation index. *P < 0.05, **P < 0.01, one sample t test and two-way ANOVA followed by Tukey’s post hoc test (Gq-Saline versus CNO: q(39) = 4.8, p = 0.008). (D) Same as (B) but quantification of the delay period between the clean air or odor presentation (Gq: q(7) = 3.37, p = 0.048; mCh: q(7) = 0.36, p = 0.8). Arrows represent post odor delivery intervals. *P < 0.05, one-way repeated measures ANOVA followed by Tukey’s post hoc test. Data for this figure are provided in S1 Data. (TIF) [file pbio.3003374.s006.tif]

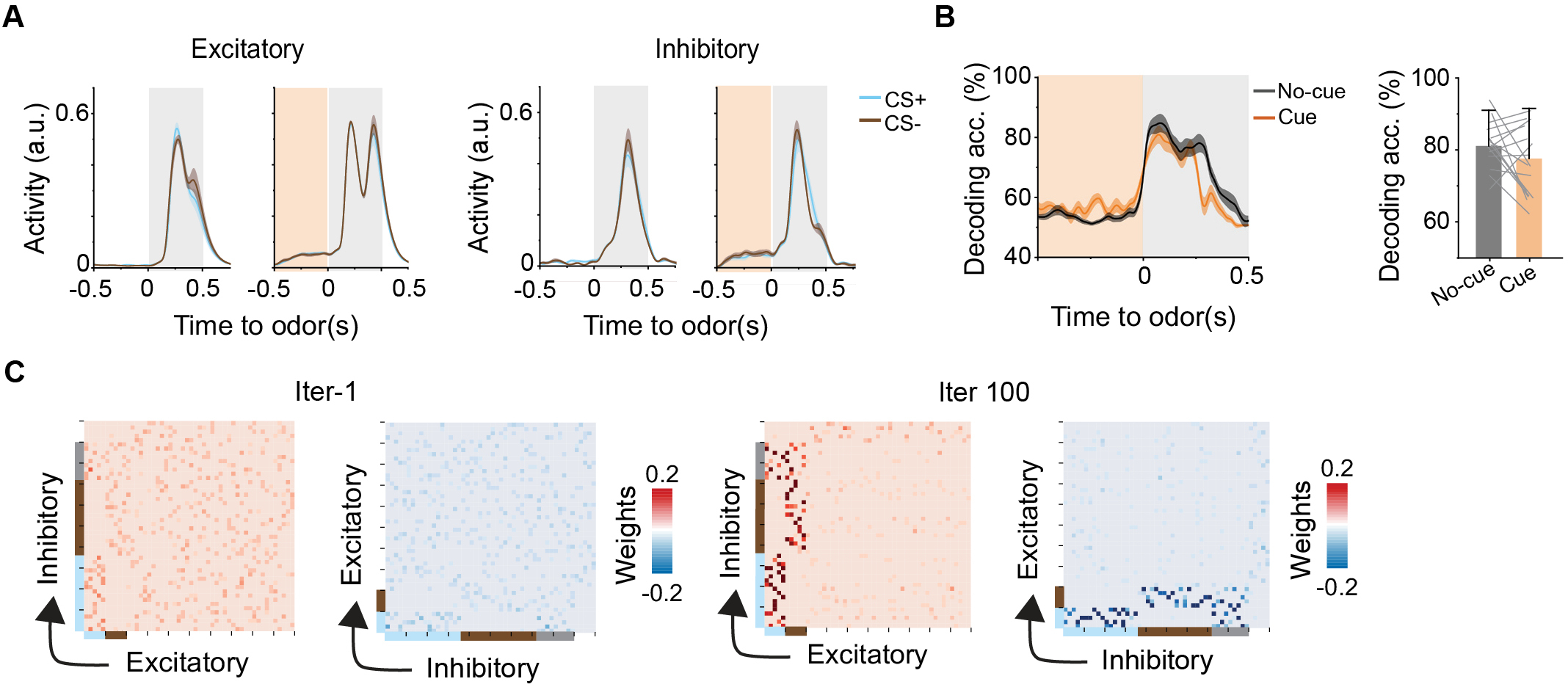

Supplement: S7 Fig — (A) Average activity of excitatory (left) and inhibitory clusters (right) to odor stimulation without implementing learning rules. (B) Decoding accuracy of stimulus identity with or without the attention cue in the absence of valence-based learning. Top–down attention was modeled from t = −0.5 to 0 s, input signal and top–down odor bias was modeled from t = 0 to 0.5 s. Right: Accuracy in decoding with or without attention cue for 16 pairs of input patterns. One-way repeated measures ANOVA followed by Tukey’s post hoc test (q(15) = 2.16, p = 0.14). (C) Changes in synaptic weights across excitatory and inhibitory neurons after training. Blue and brown shading show CS+ and CS− inputs respectively, gray shading shows inhibitory neurons common to both inputs. Data for this figure are provided in S1 Data. (TIF) [file pbio.3003374.s007.tif]
